# Supplementary material for: Soft-matter-induced orderings in a solid-state van der Waals heterostructure
Source: Nat Commun. 2025 Mar 10;16:2359. doi: 10.1038/s41467-025-57690-0 (PMC11893783; doi:10.1038/s41467-025-57690-0)
Supplement: Supplementary file 1 — Supplementary Information [file 41467_2025_57690_MOESM1_ESM.pdf]

Supplementary Information for

Soft-matter-induced orderings in a solid-state van der Waals heterostructure

Outlines

1. Schematic illustration of the design workflow of the square DNA origami nanostructure for tessellation.
2. Schematic images of the process for depositing DNA origami films on a SiO_2 (300 nm)/Si^{++} wafer.
3. DNA origami films deposited on different hard substrates.
4. DNA origami films deposited on Al_2O_3 and HfO_2 films.
5. Nanofabrication compatibility tests.
6. The optical microscope image of DNA origami films on SiO_2 (300 nm)/Si^{++} wafers.
7. Reflection spectra of DNA origami film and monolayer graphene on SiO_2 (300 nm)/Si^{++} wafers.
8. The fitted refractive index of DNA origami film.
9. Supplementary Note 1.
10. Young's modulus test of a suspended DNA film.
11. Thermal stability of DNA origami.
12. The angle identification of twisted double-layer DNA tessellations through fast Fourier transformation (FFT).
13. Schematic drawings of the comparison of trivial charge-impurity doping and the superlattice effect on graphene.
14. The workflow of the DNA-superlattice-based device fabrication.
15. The COMSOL simulation of superlattice-induced periodic potential.
16. Landau fan map (R_{xy}) of a typical device with DNA superlattice.
17. Sample-S15 for reproducibility.
18. Reproducibility of the observed additional Landau fan.
19. Hexagonal DNA origami 2D film (wavelength = 36 nm) and Sample-S23.
20. The influence of the metal layer thickness beneath Al_2O_3 on the visibility of DNA origami 2D film.
21. Calculated band structure and density of states (DOS) for graphene/DNA superlattice.
22. Supplementary Note 2.
23. The Landau fan of DNA/graphene heterostructure for the Sample-S18 across multiple regions.
24. Supplementary Table 1. Core staples of M13mp18 square DNA origami tile.
25. Supplementary Table 2. Edge staples of M13mp18 square DNA origami tiles.
26. Supplementary Table 3. Reinforcing edge staples of M13mp18 square DNA origami tiles.
27. Supplementary Table 4. Core staples of p3548 regular hexagonal DNA origami tiles.
28. Supplementary Table 5. Edge staples of p3548 regular hexagonal DNA origami tiles.

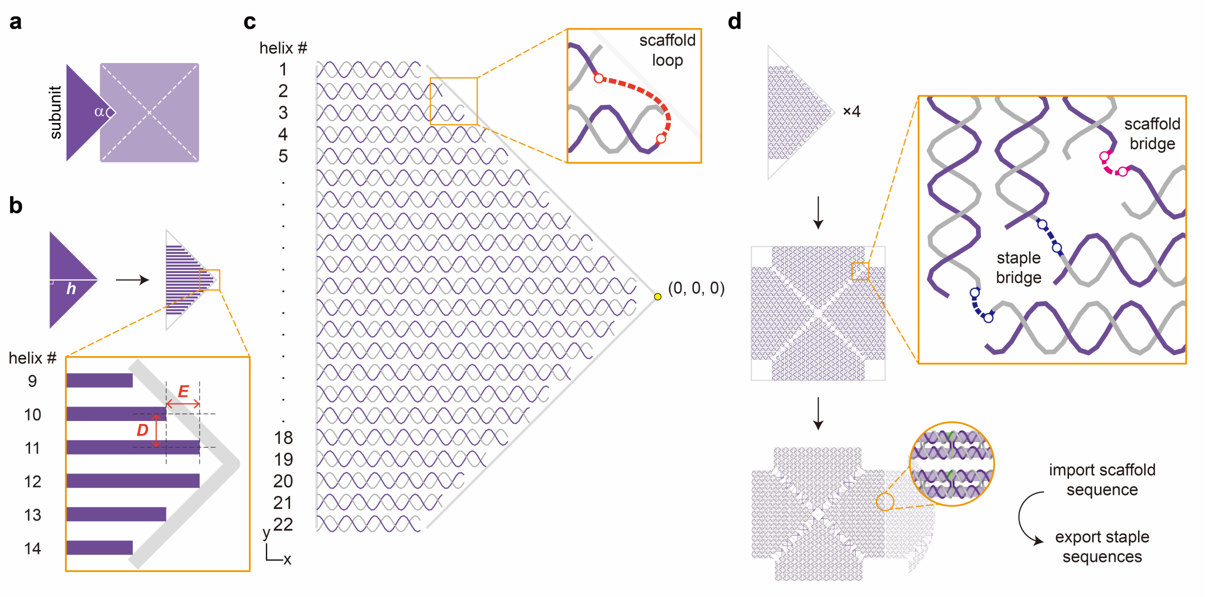

Supplementary Figure 1. Schematic illustration of the design workflow of the square DNA origami nanostructure for tessellation. (a) The square DNA origami nanostructure is composed of four copies of a repeating subunit equivalent to an isosceles right triangle with a 90° vertex angle (α). (b) The subunit can be depicted as an even number (22, in this case) of regularly-spaced line segments perpendicularly aligned at the edge opposite to α. Each line segment is a simplified representation of a DNA double helix. Inset: Two key design parameters, interhelical distance (D) and helical extension (E), are defined to describe the distance between neighboring DNA helices and their difference in length, respectively. (c) Line segments are converted into double-stranded DNA in the Euclidean space based on the structural attributes of B-form DNA. The (0, 0, 0) point is assigned to the vertex of the subunit. The strands for creating the scaffold are purple-colored, while their complementary strands for creating staples are gray-colored. The two terminal nucleotides of neighboring purple strands are connected by single-stranded linkages (termed scaffold loops). Inset: Illustration of an example scaffold loop connecting the purple strands in the second and third helices. (d) Four copies of the subunit are joined together to obtain the prototypic origami nanostructure, in which neighboring subunits are bridged by scaffold and staple bridges (inset). The design is finalized using Tiamat, a design software tool for designing DNA nanostructures from scratch, with sequence of the scaffold strand assigned. The matching rule governing the interaction between monomeric DNA origami nanostructures is defined by prescribing base paring and stacking interactions at their edges (inset). Sequences of the staple strands are generated as the output for synthesis (listed in the Supplementary Tables 1-5).

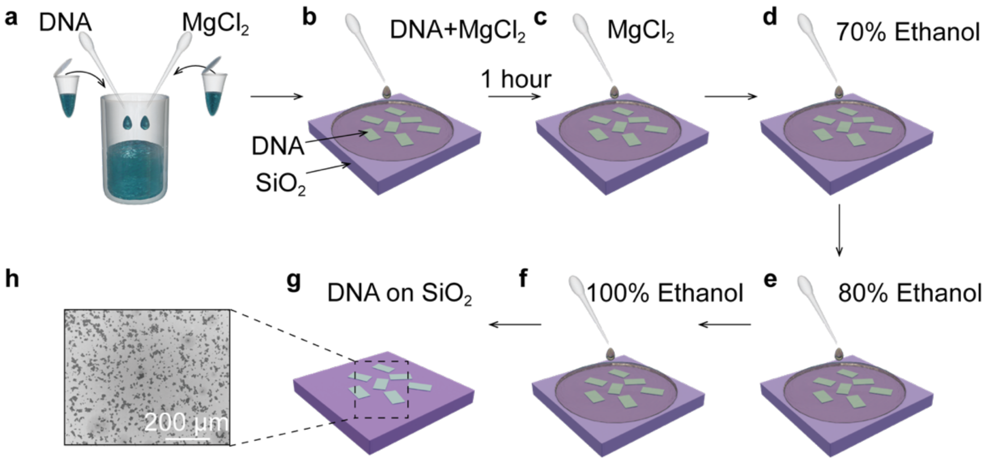

Supplementary Figure 2. Schematic images of the process for depositing square DNA origami films on a SiO_2 (300 nm)/Si^{++} wafer. (a) Dilution of the DNA solution. The DNA solution were diluted by mixing with $MgCl_2$ solution (10.5 mM) at a ratio of 1:39. (b) Dropping of the diluted DNA solution. 20 μL DNA solution was dropped onto O^2 plasma-cleaned SiO_2 substrate and held for 1 hour. (c) Rinsing of the DNA-deposited substrate with $MgCl_2$ solution (10.5 mM). The $MgCl_2$ solution was dropped onto the substrate and then the substrate was blown dry with N_2. (d-f) Rinsing of the DNA-deposited substrate with 70%, 80%, and 100% ethanol solutions. Ethanol solutions of different concentrations were dropped onto the substrate and then the substrate was blown dry with N_2. (g) Dry DNA origami films on SiO_2 substrate. (h) SEM image of DNA origami films. Different from the square superlattices, hexagonal superlattices were diluted by mixing with 1×TAE-Mg buffer solution (containing 12.5 mM Mg^{2+}) at a ratio of 1:3 before deposition, 3 μL of the diluted DNA solution were dropped onto the substrates and held for 30 minutes. Then the DNA films were gently rinsed and blown dry with the same methods as the previous square superlattice, except that $MgCl_2$ was replaced with 1× TAE-Mg buffer solution.

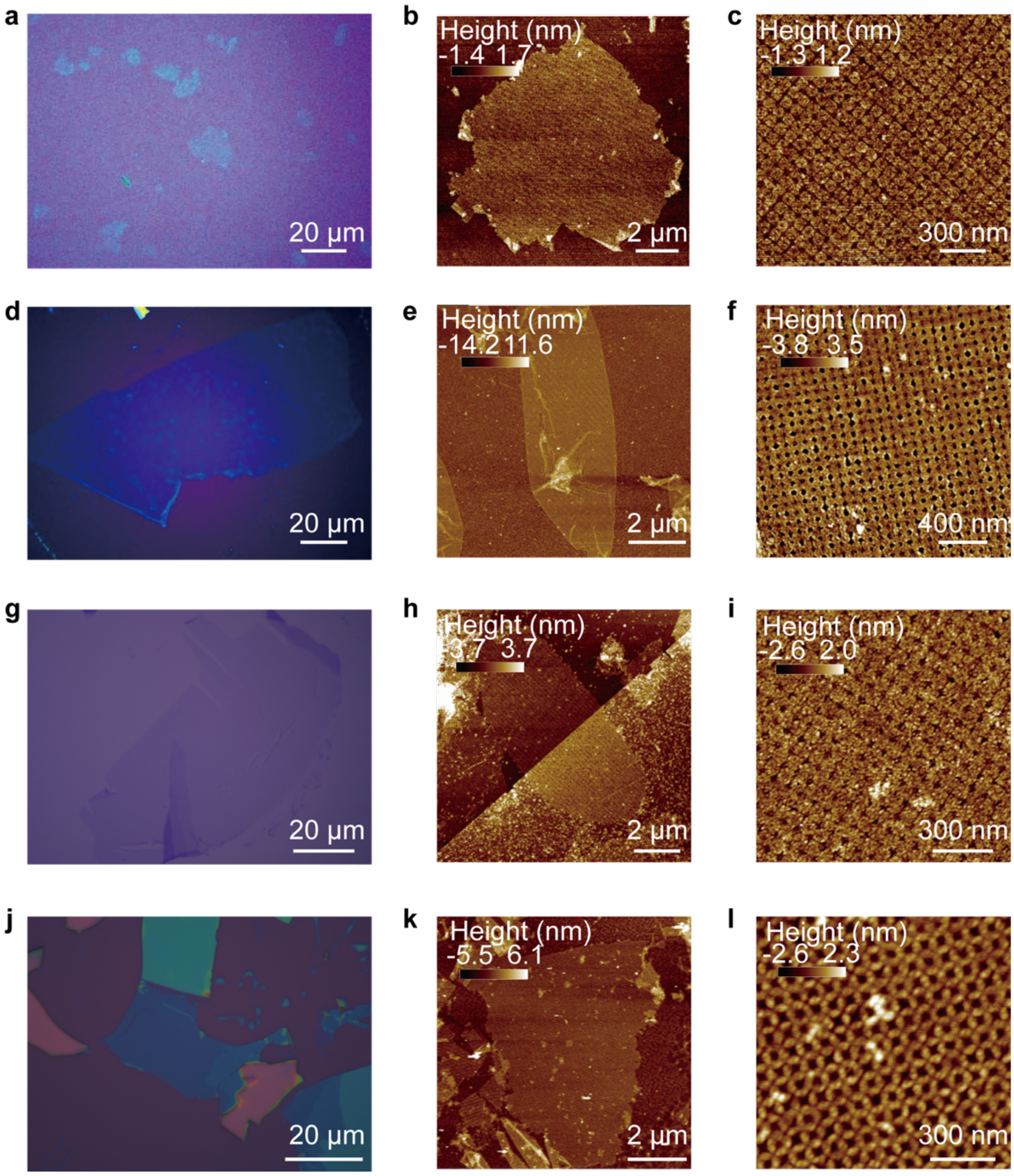

Supplementary Figure 3. DNA films deposited on different hard substrates. Optical, AFM and detailed AFM images of the DNA origami films deposited on a SiO_2 (300 nm)/Si^{++} wafer (a-c), h-BN (d-f), monolayer graphene (g-i) and mica (j-l) after being dried in air.

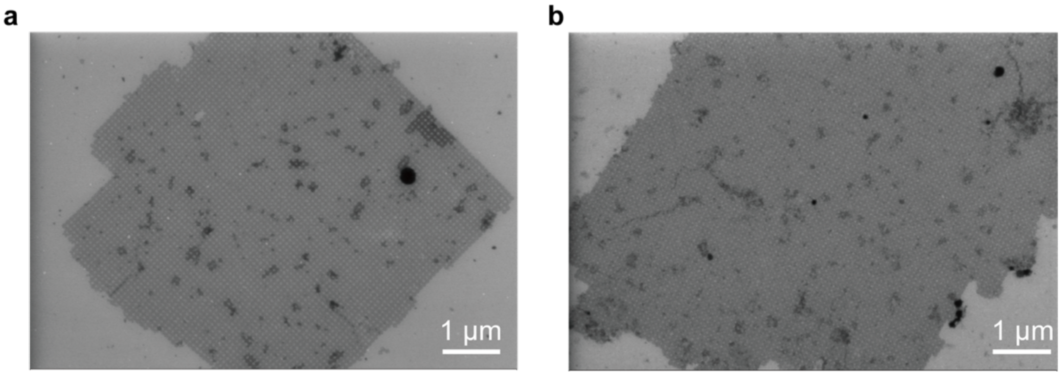

Supplementary Figure 4. DNA films deposited on Al_2O_3 and HfO_2 films. (a) SEM image of the DNA origami film deposited on a Al_2O_3 film. (b) SEM image of the DNA origami film deposited on HfO_2 film.

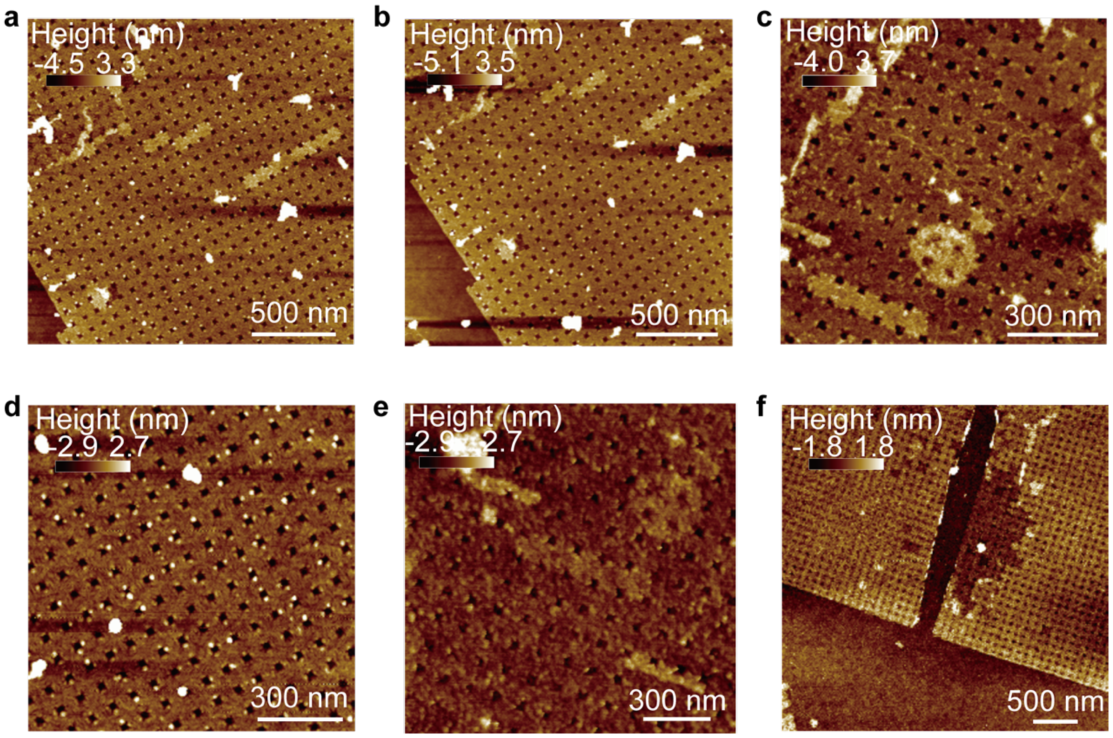

Supplementary Figure 5. Nanofabrication compatibility tests. (a-d) AFM image of a DNA film after being sequentially soaked in ethanol solution (a), isopropanol solution (b), acetone solution (c), and trichloromethane solution (d) for 2 minutes, and dried in air for each step. (e) AFM image of a DNA film after being kept in vacuum at 320 °C for 20 minutes and cooled to room temperature in vacuum. (f) DNA cleavage achieved through AFM's anodizing.

In the community of biosynthesis, DNA origami assemblies are often deposited on mica and characterizaed mostly in liquid phases, which is very different when in the cases of solid state nanoelectronic devices. Here, in our work, the primary objective of presenting Supplementary Figures 3–5 is to demonstrate the compatibility of soft matter DNA with various hard substrates, as well as the solvents used in nanofabrication processes – since the solid states nanoelectronics are usually built on typical substrates such as silicon oxides. To achieve this, we deposited 2D DNA origmai flakes from solution onto different substrates, followed by drying, and examined its morphology and periodicity using Optical Microscopy (OM), Atomic Force Microscopy (AFM), and Scanning Electron Microscopy (SEM).

By evaluating how well the intrinsic periodicity of DNA was maintained on various substrates—including h-BN, graphene, SiO_2 (300 nm)/Si^{++} wafer, Al_2O_3, HfO_2, and mica (Supplementary Figures 3–4)—we identified Al_2O_3 as the most suitable substrate for subsequent heterostructure fabrication. Notably, Al_2O_3 deposited via the Atomic Layer Deposition (ALD) method provides a large-scale, uniform, and insulating film, making it an ideal choice for preserving DNA's periodic structure.

Additionally, we tested the stability of DNA’s periodicity by immersing it in different solvents commonly used in device fabrication (Supplementary Figure 5). These experiments confirmed that the solvents do not cause severe damages to DNA’s periodic structure, ensuring its compatibility with the following up nanofabrication processes. These findings are crucial in selecting appropriate substrates and solvents that preserve the DNA superlattice, providing references for possible integration into heterostructures with hard materials like graphene.

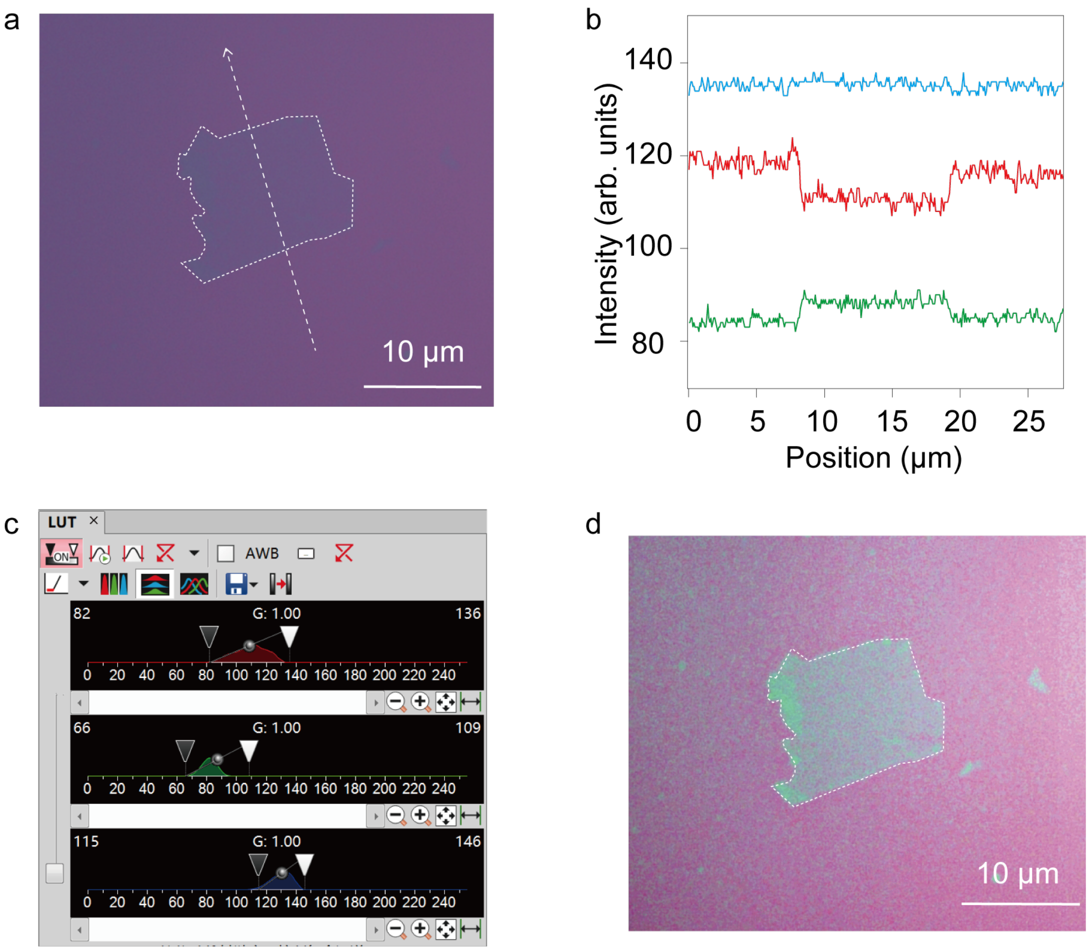

Supplementary Figure 6. The optical microscope image of DNA origami film on SiO_2 (300 nm)/Si^{++} wafer. (a) Original optical image of the DNA origami film on SiO_2 wafer taken with Nikon-LV-ND-100. (b) The RGB intensities of the DNA origami film. (c) Modulation of the red, green, and blue channels on the optical microscope software. (d) The optical image of DNA origami film after modulating three channels.

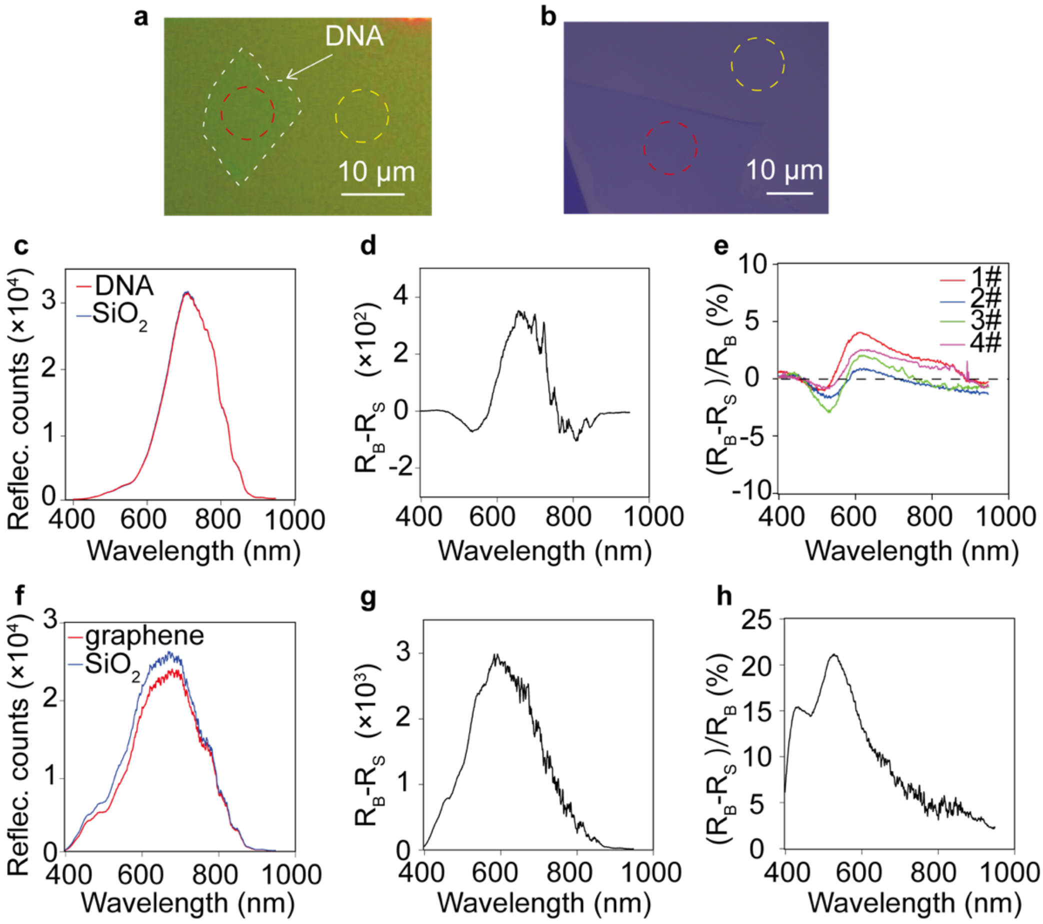

Supplementary Figure 7. Reflection spectra of DNA superlattice and monolayer graphene on SiO_2 (300 nm)/Si^{++} wafers. (a) Optical microscope image of a DNA superlattice used for reflectance measurement. (b) Optical microscope image of a monolayer graphene used for reflectance measurement. The red circles and yellow circles in (a) and (b) are the light spot positions when testing sample (DNA film or graphene) and background SiO_2 (300 nm)/Si^{++} wafer respectively. (c) Reflectance spectra of a DNA superlattice and SiO_2 background. (d) The reflectance difference between the background and the DNA superlattice, R_B-R_S. (e) The relative reflectance difference between the background and the DNA superlattice, (R_B-R_S)/ R_B. (f-h) The reflectance spectra (f), reflectance difference (g) and relative reflectance difference (h) of the monolayer graphene on a SiO_2 (300 nm)/Si^{++} wafer.

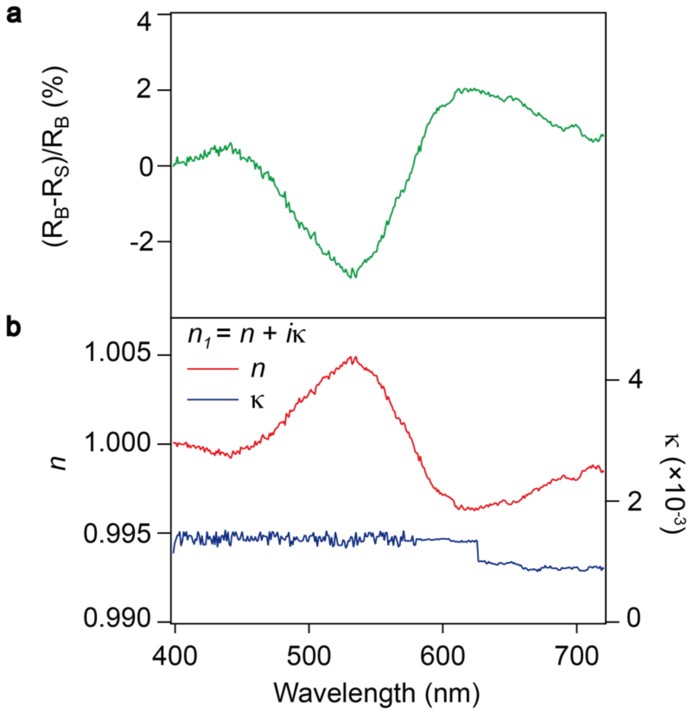

Supplementary Figure 8 (a) The renormalized reflectance difference, defined as $(R_B - R_S)/R_B$, where R_B and R_S are the reflectance measured at the background and the sample areas, respectively, for the 2D DNA film placed onto a SiO_2 (300 nm)/Si^{++} substrate, as a function of the wavelength of the perpendicularly incident light beam. (b) The fitted refractive index $n_1 = n + ik$ of DNA, where the red (blue) line represents the real part n (imaginary part k) of n_1.

Supplementary Note 1.

To explain the observed variations in light reflection across different wavelength, we follow the approach outlined in published paper[1] . We consider the case of normal incidence of light from air (with refractive index $n_0 = 1$) onto a trilayer structure consisting of DNA, SiO_2, and Si. The Si layer is assumed to be semi-infinite and characterized by a complex refractive index n_3, which is wavelength (λ) dependent[2]. The SiO_2 layer has a thickness d_2 = 300 nm and a refractive index n_2, which is purely real and also wavelengh(λ) dependent [3, 4]. The DNA layer, with thickness d_1, is considered to be 2 nm, as shown in Figure 3c of our main text. The complex refractive index n_1 of DNA is treated as a fitting parameter in our calculations.

With this configuration, we derive the reflection coefficients and the reflected intensity.

The reflection coefficients are given by:

$$r_1 = \frac{n_0 - n_1}{n_0 + n_1} \tag{1}$$

$$r_2 = \frac{n_1 - n_2}{n_1 + n_2} \tag{2}$$

$$r_3 = \frac{n_2 - n_3}{n_2 + n_3} \tag{3}$$

And the phase shifts are defined as:

$$\Phi_1 = \frac{2\pi n_1 d_1}{\lambda} \tag{4}$$

$$\Phi_2 = \frac{2\pi n_2 d_2}{\lambda} \tag{5}$$

And the total reflected intensity $I\,(n_1)$ is defined as

$$I\,(n_1) = \left| r_1 e^{i(\Phi_1+\Phi_2)} + r_2 e^{-i(\Phi_1+\Phi_2)} + r_1 r_2 r_3 e^{i(\Phi_1-\Phi_2)} + r_2 r_3 e^{-i(\Phi_1-\Phi_2)} \right|^2 \tag{6}$$

, which accounts for the reflections from each layer and the phase shifts that occur as light traverses the different materials.

Then the contrast C is defined as the relative intensity of reflected light when the DNA is present (n_1) compared to when $n_1 = 1$ (no DNA, i.e., $n_1 = n_0 = 1$). It is calculated as:

$$C = \frac{I_{n_1=1} - I_{n_1}}{I_{n_1=1}} \tag{7}$$

where $I_{n_1=1}$ is the reflected intensity when $n_1 = 1$ (in the absence of DNA), and I_{n_1} is the reflected intensity for the current value of n_1.

This approach allows us to model the light reflection shown in Figure 2d of our main text, which corresponds to the light contrast C. The fitted n_1 values are presented in Supplementary Figure 8b, where the red represents the real part of n_1, denoted as n, and the blue represents the imaginary part of n_1, denoted as k.

The calcualted real part n of the DNA refractive index, as obtained through fitting the above formulas, shows that n has relatively lower values in the wavelength rage of ~ 600–700 nm (red visible light region). Correspondingly, the observed optical contrast changes in OM images (shown in Figure 2c of the main text) reveals an reduction in the reflected intensity in the red light range where DNA is present. In contrast, the real part of n is relatively larger in the green light region (~ 500–600 nm), and the observed optical contrast changes in OM images in this range shows a enhancement in the reflected intensity. The wavelength-dependent behavior of the DNA refractive index may offer insight into the mechanism by which the DNA superlattice interacts with light in the relevant spectral range.

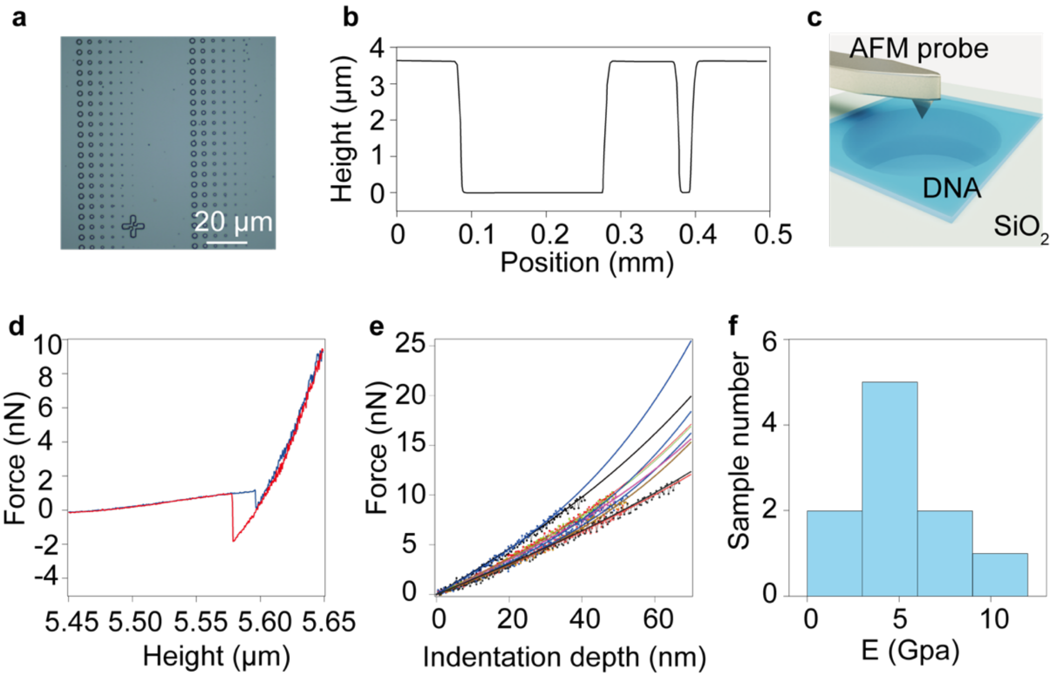

Supplementary Figure 9. Young's modulus test of a suspended DNA film. (a) The holely substrate with diameters from 500 nm to 5 µm. (b) The depths of holes in (a). (c) Schematic diagram of an AFM probe exerting compressive stress on a suspended DNA film. (d) The typical force curve obtained by AFM. The maximum force of the probe pressing on the suspended film is 10 nN. (e) Force curve fitting results at 10 different positions. Lines with markers are experimental data and solid lines are fitting results. (f) Histogram of the DNA film's Young's modulus obtained from the fitting results in (e).

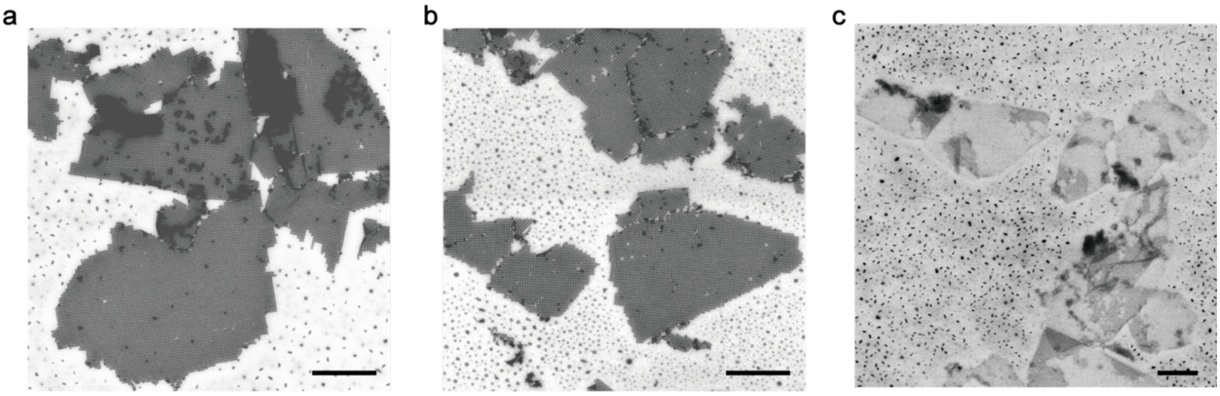

Supplementary Figure 10. Thermal stability of DNA origami. SEM images of DNA origami after heating at 180°C for 30 minutes in pure oxygen (a), pure nitrogen (b), and humid air (c). The scale bars in (a-c) are 2 μm.

It is found that when there is absence of moisture (in Supplementary Figure 10a pure O_2, or in Supplementary Figure 10b pure N_2 enviroment, for instance), heating at 180 ºC for 30 min do not affect too much the morphology of DNA origami structures. However, stark contrast can be seen in the presence of moisture. As can be seen in Supplementary Figure 10c, clear degradation of the tessellation morphology is observed after heating the DNA origami in humid air for 30 min. Notice that each image was taken by SEM scan of independent samples, to avoid electron beam exposure prior to heating tests.

The thermal stability of DNA self-assembled structures in aqueous solutions has been extensively studied, with melting temperatures not exceeding 80 degrees Celsius[5]. The primary reason for structural disruption is the intensification of molecular thermal motion, where hydrogen bonds and base stacking that maintain the structural morphology are insufficient to restrain the molecules.

A possible hypothesis is that in a pure gas phase, even if molecules undergo thermal motion, the absence of a diffusion medium makes it difficult for the molecules to diffuse and lose their morphology. However, humid air provides a diffusion medium, which may cause the molecules to lose their structural morphology due to diffusion.

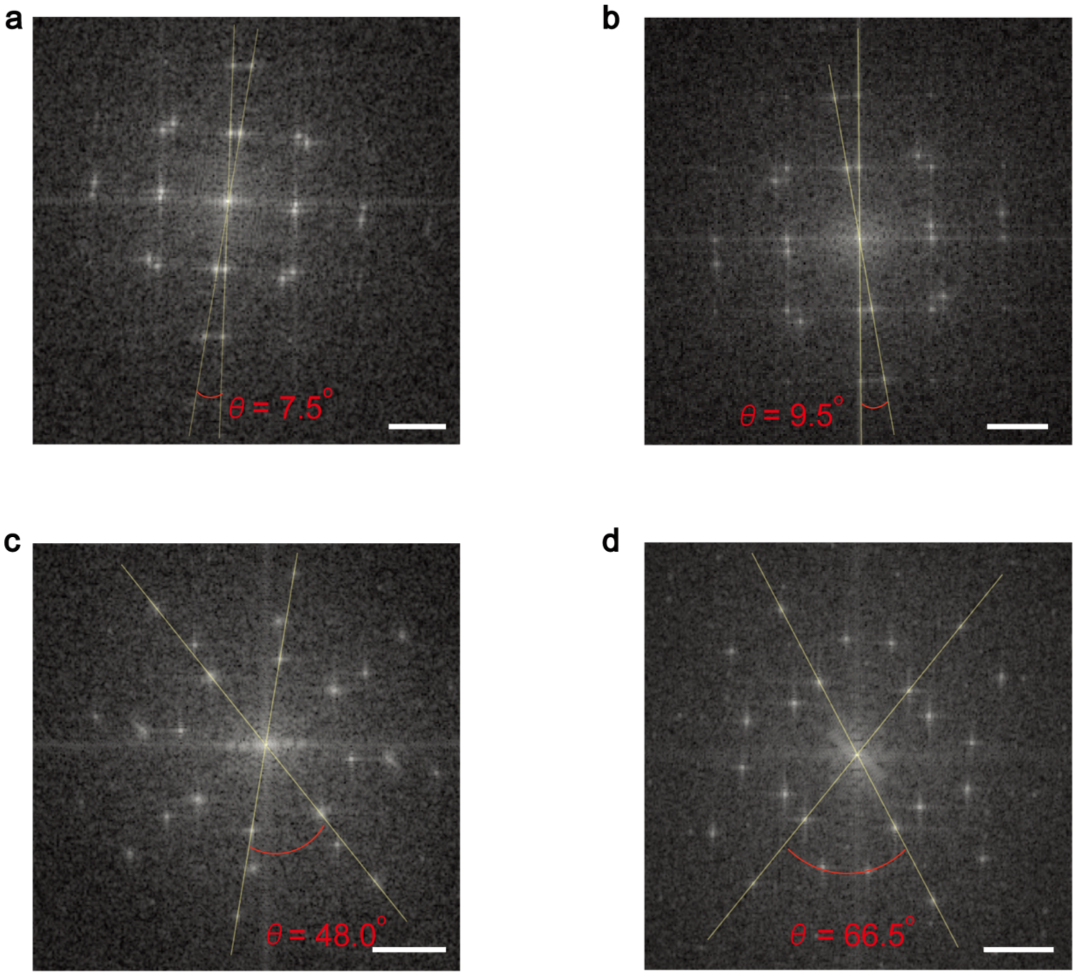

Supplementary Figure 11. The angle identification of twisted double-layer DNA tessellations through fast Fourier transformation (FFT). In (a) to (d), the two sets of yellow lines correspond to the upper and lower periodic DNA tessellations. The angle between these two sets of lines represents the twisted angle between the two DNA layers. The scale bars in (a)-(d) are 10 μm^{-1}.

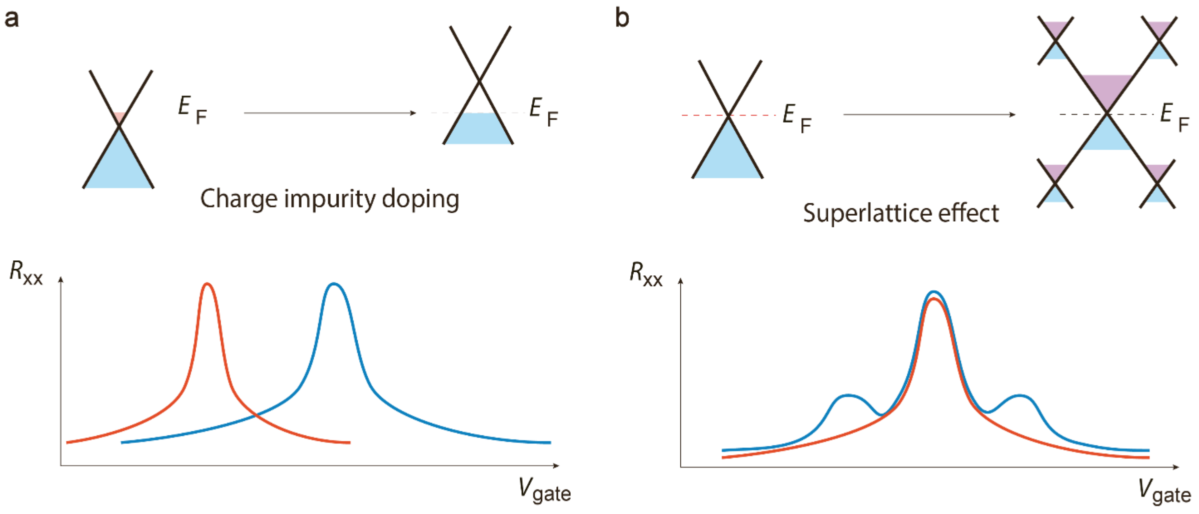

Supplementary Figure 12. Schematic drawings of the comparison of trivial charge-impurity doping and the superlattice effect on graphene. (a) Trivial charge-impurity doping and the consequence of Fermi level shifting of graphene without band reconstruction. (b) Band reconstruction of graphene due to the existence of an artificial superlattice. Two side peaks can be seen due to the formation of additional minima in the density of states (which yield a resistance maximum accordingly, in the transport behaviors).

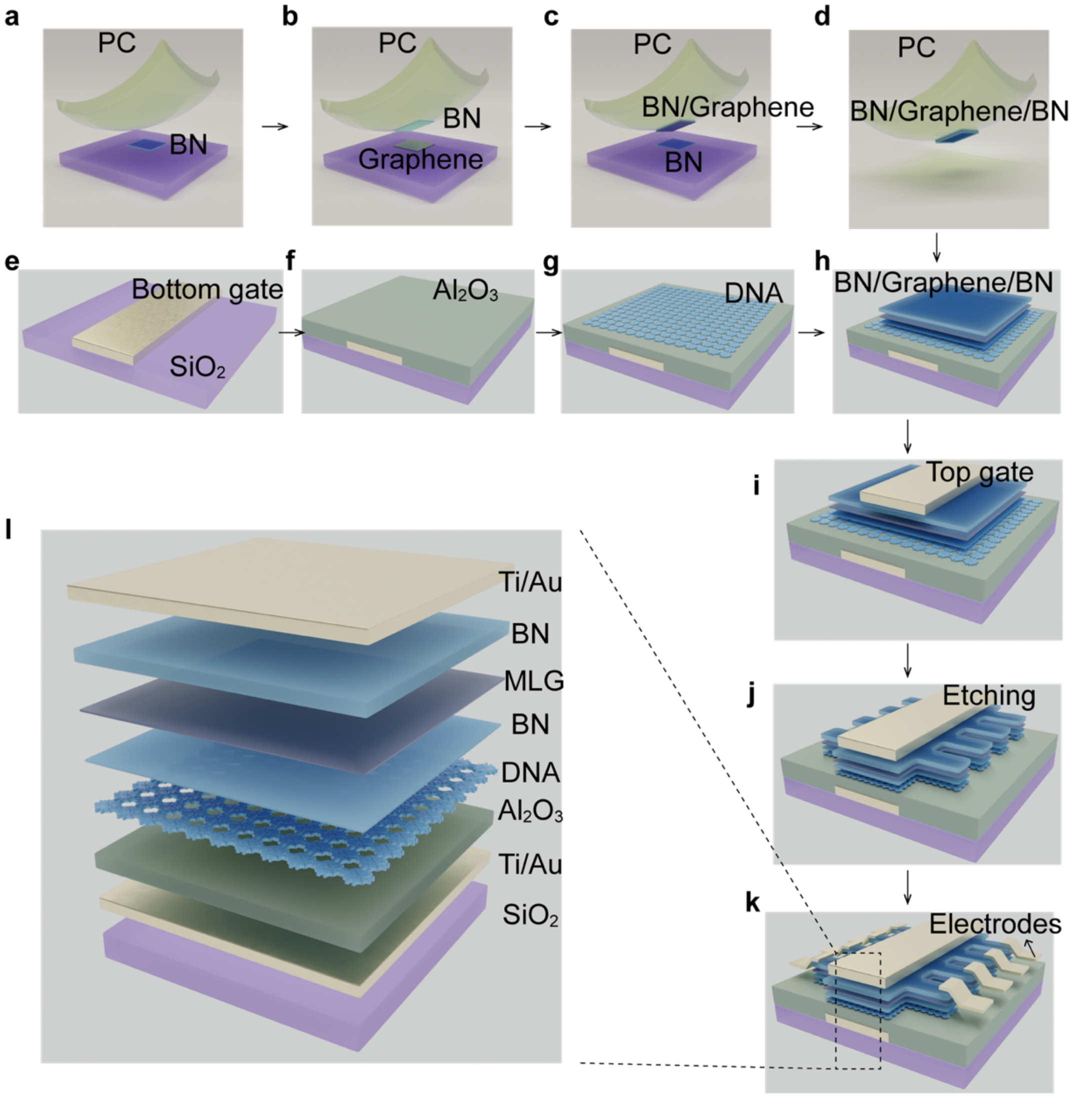

Supplementary Figure 13. The workflow of the DNA-superlattice-based device fabrication. (a-d) Schematic images of the process for picking up the BN-encapsulated graphene with PC (Polycarbonate). (e) The bottom gate (Ti/Au) prepared on a SiO_2 (300 nm)/Si^{++} wafer. (f) 15-nm Al_2O_3 dielectric layer deposited on the bottom gate via atomic layer deposition. (g) DNA film deposited on the Al_2O_3 dielectric layer. (h) The BN/graphene/BN transferred on DNA film. (i) Top gate deposited on the stack via electronic beam evaporation. (j) Hall bars obtained by reactive ion etching. (k) Schematic image of the final device. (l) The vertical layout of the device.

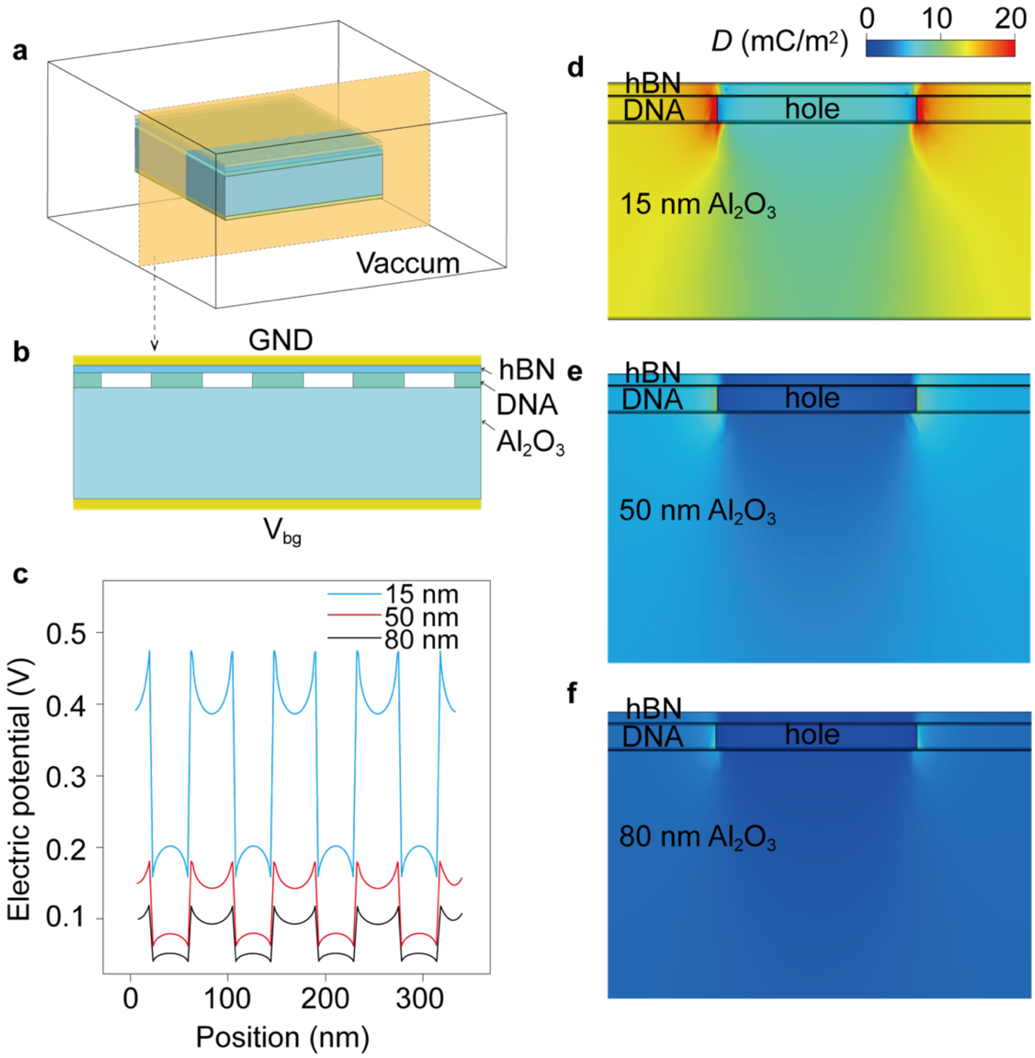

Supplementary Figure 14. The COMSOL simulation of superlattice-induced periodic potential. (a) The model used in COMSOL simulation. A five-layer structure (GND/BN/DNA/ Al_2O_3/V_{bg}) was constructed and placed in a vacuum. (b) Cross section of the five-layer structure. (c) The periodic electric potential formed on the upper surface of DNA superlattice with different Al_2O_3 thickness. (d-f) Displacement electric field (*D* distributions around the hole in vertical direction with 15 (d), 50 (e), and 80 nm (f) Al_2O_3 under the DNA superlattice.

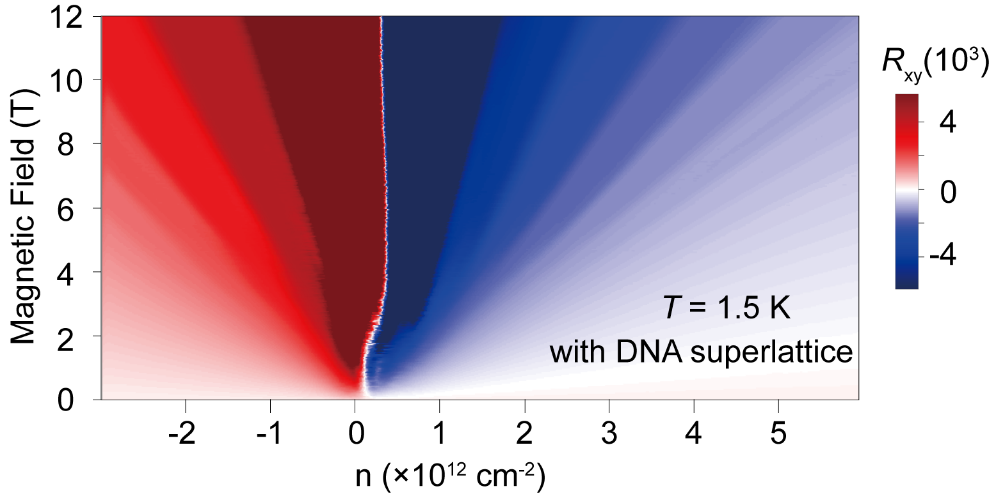

Supplementary Figure 15. Landau fan map (R_{xy} recorded in the parameter space of magnetic field B and carrier density n) of the same device with DNA superlattice in Figure 4 (d).

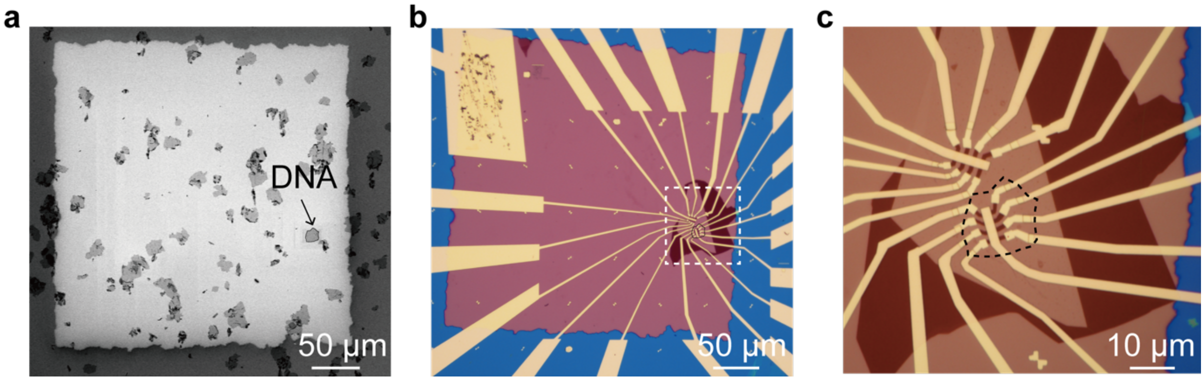

Supplementary Figure 16. Sample-S15. (a) SEM image of the DNA origami film on top of the pre-patterned bottom Au gate covered by 10 nm HfO_2. (b) Sample-S15 fabrication of a typical vertical heterostructure of h-BN (21.4 nm)/graphene/h-BN (0.73 nm) on top of the DNA film in (a). (c) Zoom-in scan of the white boxed area in (b). The DNA film is outlined by the black dashed line in (a-c). The adjacent part not outlined is the device without DNA film.

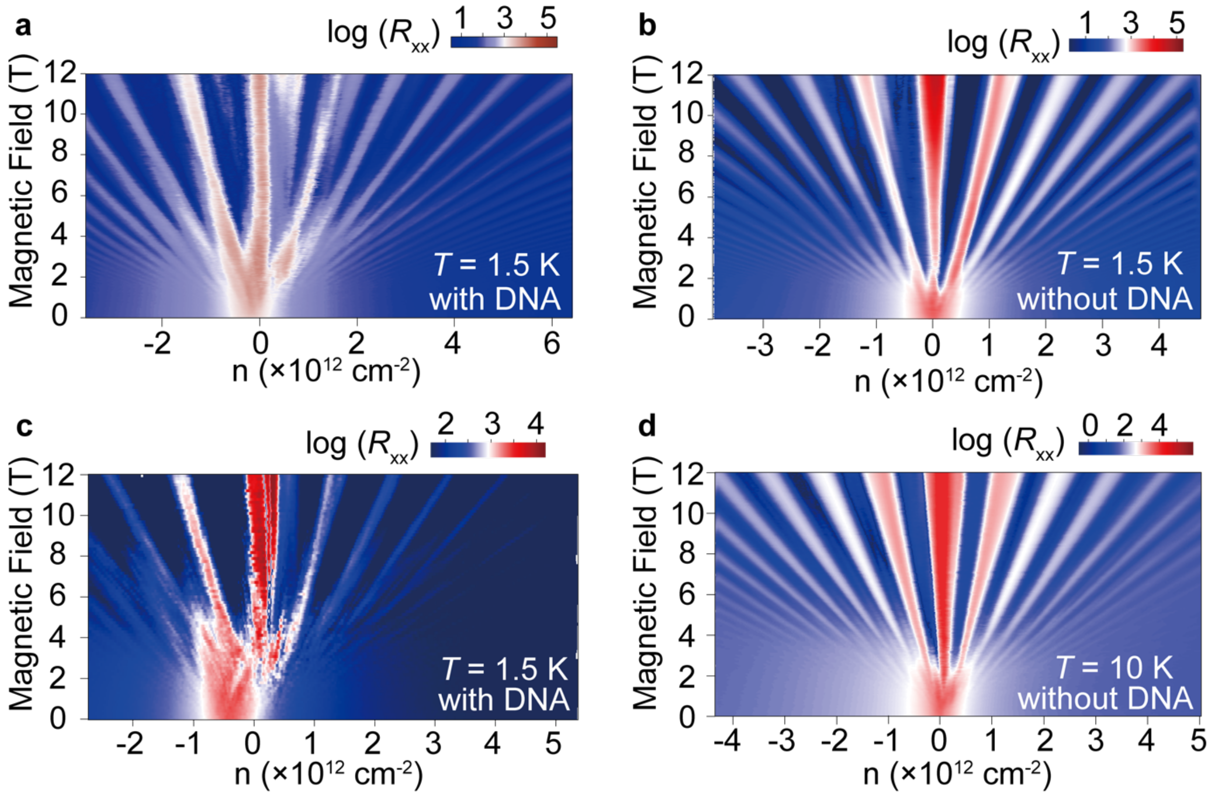

Supplementary Figure 17. Reproducibility of the observed additional Landau fan. (a) Landau fan map (R_{xx}) of Sample-S18 with DNA superlattice. It's also shown in Fig. 4d in the main text. (b) Landau fan map (R_{xx}) of Sample-S18 without DNA superlattice. (c) Landau fan map (R_{xx}) of Sample-S15 with DNA superlattice as shown in Supplementary Figure 16. (d) Landau fan map (R_{xx}) of Sample-S15 without DNA superlattice.

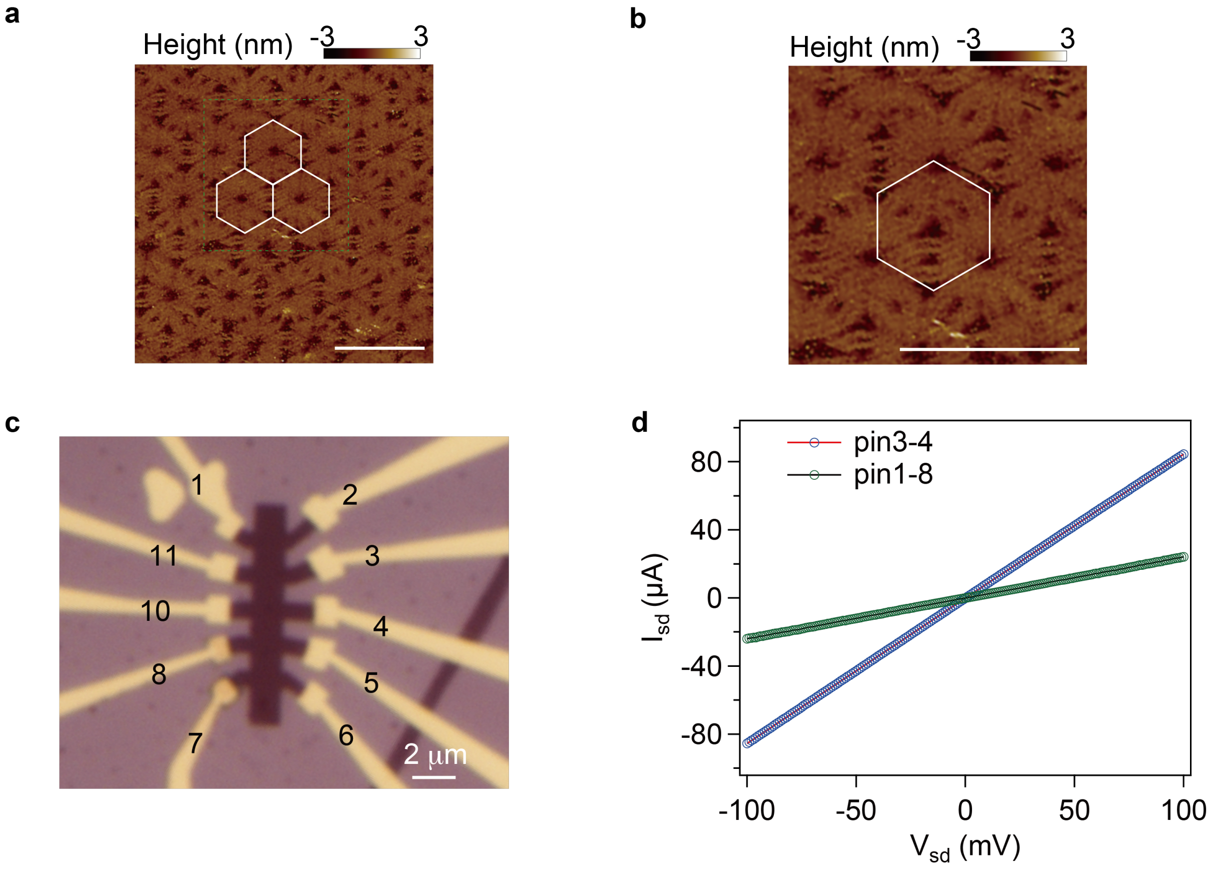

Supplementary Figure 18. **Hexagonal DNA origami 2D film (wavelength = 36 nm) and Sample-S23.** (a) AFM characterization of the 36 nm hexagonal DNA origami 2D film. (b) Zoomed-in scan of the area outlined by the green dashed box in (a). The scale bars in (a) and (b) are 100 nm. (c) Optical image of the device (Sample-S23) and schematic of the measurement setup.

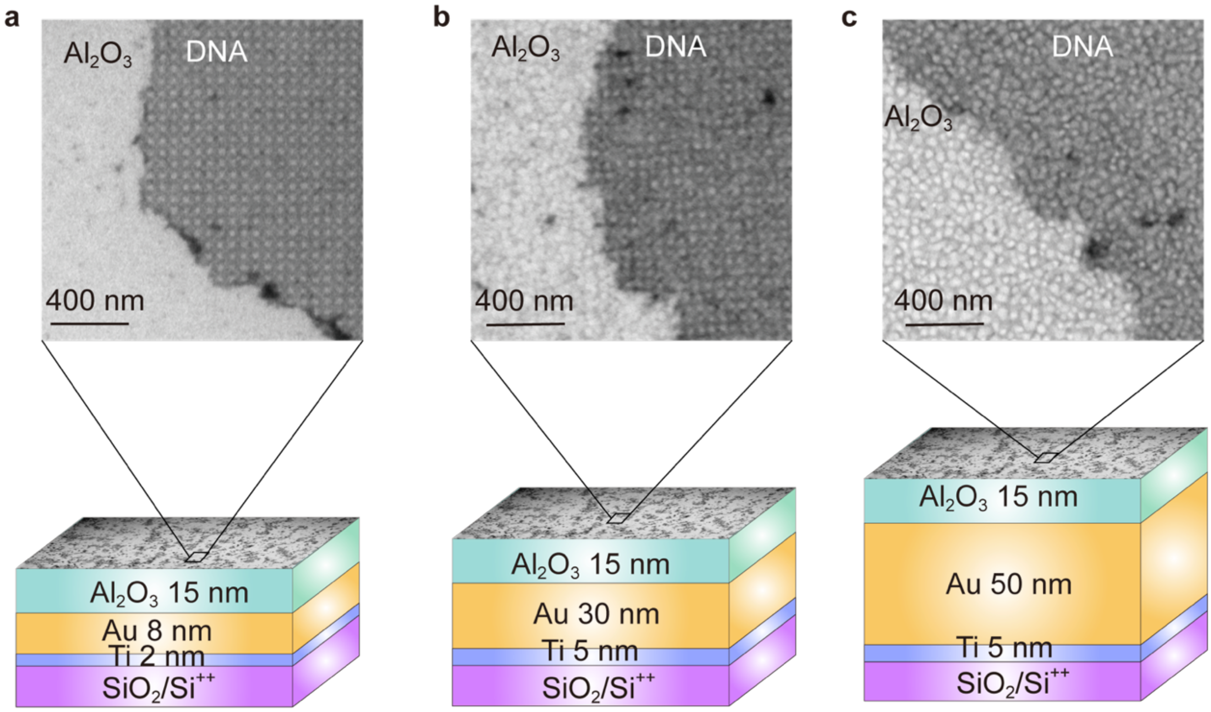

Supplementary Figure 19. The influence of the metal layer thickness beneath Al_2O_3 on the visibility of DNA origami. SEM images of DNA origami 2D film deposited on Al_2O_3 films with buried metal gate electrodes of Au/Ti-8/2 nm (a), Au/Ti-30/5 nm (b), and Au/ Ti-50/5 nm (c). The schematic diagrams at bottom of each SEM images illustrate the thickness of the buried metal electrodes.

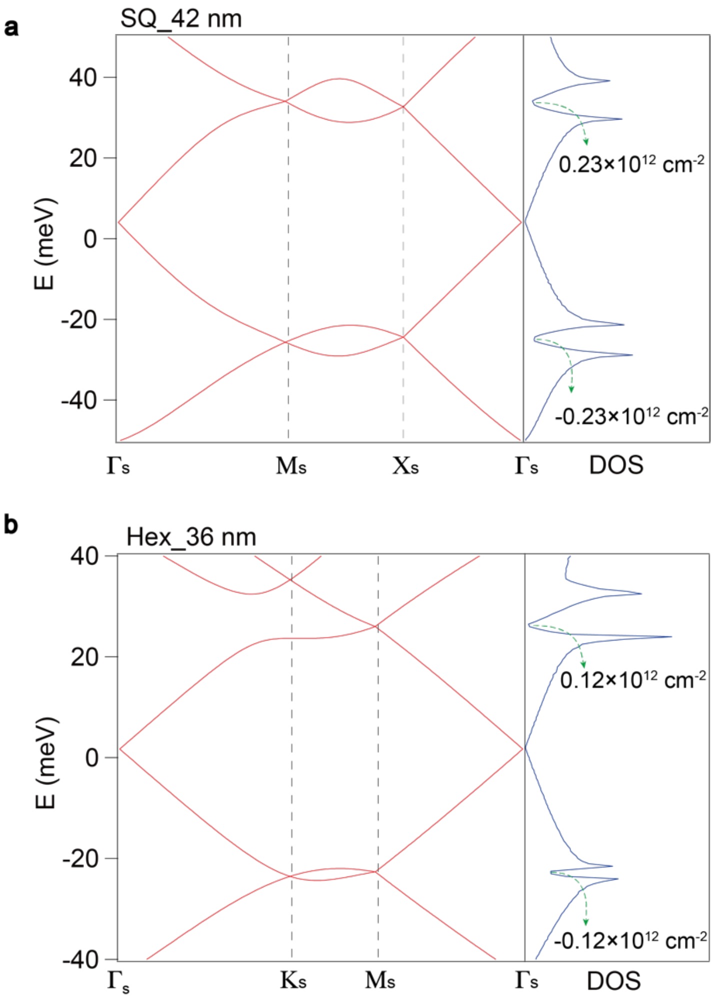

Supplementary Figure 20. Calculated band structure and density of states (DOS) for graphene under the influence of an external potential induced by the DNA film presented in square (a) or hexagonal (b) superlattice. The calculated electron and hole doping concentration are indicated in the energy levels corresponding to the minimum values of the DOS.

Supplementary Note 2.

Using previously reported method in similar superlattice system realized by nano-sized etching using focus ion beam[6], we construct the following Hamiltonian derived from a continuum model to illustrate the band structure and density of states (DOS) of graphene when the DNA superlattice is present

$$H_\mu(\boldsymbol{k}) = \hbar v_F \boldsymbol{k} \cdot \boldsymbol{\sigma}_\mu + U(\boldsymbol{r}) \tag{8}$$

where $\mu = \pm 1$ is the valley index respectively for **K** and **-K**, with $\boldsymbol{\sigma}_\mu = (\mu\sigma_x, \sigma_y)$ denoting Pauli matrices in the sublattice space of graphene. $\boldsymbol{k} = (k_x, k_y)$ is the 2D wavevector relative to the valley wavevector **K**/-**K**. v_F denotes the Fermi velocity and the value of the parameter is $\hbar v_F = 5.25\ \text{eV} \cdot \text{Å}$. $U(\boldsymbol{r})$ refers to the external periodic potential from the DNA superlattice, which is expanded in Fourier series truncated to include only the shortest reciprocal lattice vectors of the superlattice

$$U(\boldsymbol{r}) = V \sum_{m_1 m_2} e^{i(m_1 \boldsymbol{G}_1 + m_2 \boldsymbol{G}_2)\boldsymbol{r}} \tag{9}$$

where V is the potential amplitude. We solve the eigenvalues of the above Hamiltonian at each $\boldsymbol{k}$ momentum under a 9×9 plane-wave basis set, which gives 162 electron bands. Here we focus on the low-energy band structure at the **K** valley, as the −**K** valley structure can be derived through time-reversal symmetry. A dense 300×300 k-point grid is used to ensure the convergence in the DOS calculation. The doping concentration is numerically obtained by the following formula

$$n_{doping} = \frac{1}{N_k \Omega} \int_{E_f}^{E_0} g(E) dE \tag{10}$$

where Ω is the area of superlattice unit cell and N_k is the number of $\boldsymbol{k}$ points. $g(E)$ is the DOS at E level. E_f is the Fermi energy and E_0 is the energy corresponding to the minimum value of the DOS.

We adopt the potential amplitude $\text{V} = 36\ \text{meV}$ when the DNA film is presented in a square superlattice of side length $42\ \text{nm}$. Supplementary Figure 19a shows the band structure and DOS. Both the electron and hole doping concentration are calculated to be $0.23 \times 10^{12}\ \text{cm}^{-2}$. For the DNA film in a hexagonal superlattice of side length $36\ \text{nm}$, with the choice of potential amplitude $\text{V} = 20\ \text{meV}$, the doping concentration is calculated to be $0.12 \times 10^{12}\ \text{cm}^{-2}$ as indicated in Supplementary Figure 20b.

These simulation data are in great agreement with the experimental results.

We also note that the selection of potential amplitude is highly flexible as quite a wide range of V values yield consistent doping concentration, which underscores the robustness of the obervation in experiment.

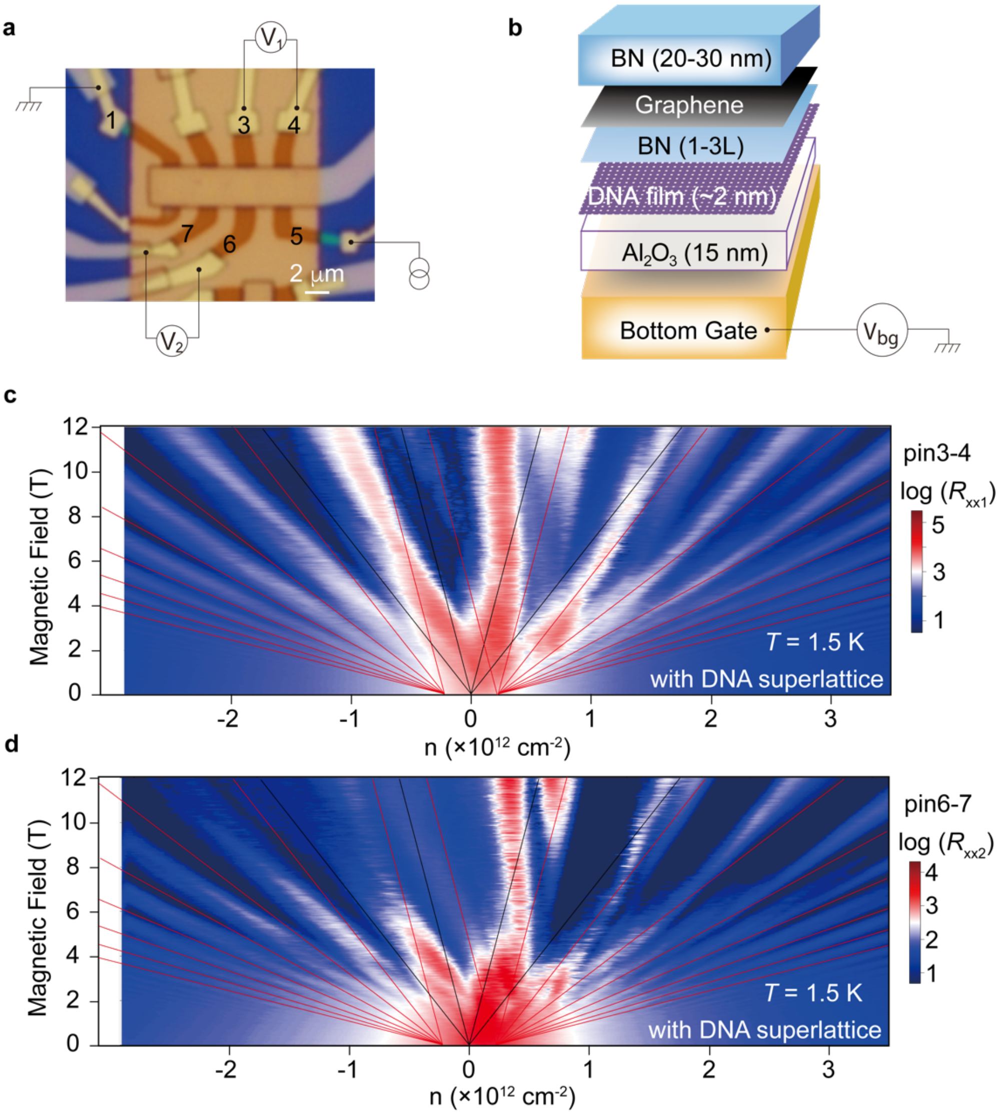

Supplementary Figure 21. The Landau fan of DNA/graphene heterostructure for the Sample-S18 across multiple regions. (a) and (b) show the optical image and schematic diagram of the graphene/DNA heterostructure, respectively. (c) and (d) display the Landau fan diagrams measured for pin3-4 and pin6-7, respectively. The solid black lines indicate the Landau levels for the main Dirac fan, while the solid red lines represent the superlattice satellite fans.

Supplementary Table1. Core staples of M13mp18 square DNA origami tile.

Name	Sequence
[SQ]_M13_core_1	GCTGAGGCTTGCAGGGAGTTAAACGAAAGACAGCATCGCATGAGGA
[SQ]_M13_core_2	AGGCAAAAGGACTAAAGACTTTTTGAACGA
[SQ]_M13_core_3	GGGTAGCAACTTTCGAGGTGAA
[SQ]_M13_core_4	AGTTTCCAAGGCACCAACCTAAGATTTGTA
[SQ]_M13_core_5	TGAGAAGTTTATCATTTTGGCTTTGAGAATAC
[SQ]_M13_core_6	GCGCAGACAACAAAGTACAACGGAAACGAAAG
[SQ]_M13_core_7	ACTAAAACACTTTTTTTTTCAGCGGAG
[SQ]_M13_core_8	TCATCGCCCATGTTACTTAGCCGTAATCTT
[SQ]_M13_core_9	AAGCGCGAGGTCAATCATAAGGGACAGGCGCA
[SQ]_M13_core_10	GATTTTGCTAAACAACTTTTTTCCCCAGCGATTATACC
[SQ]_M13_core_11	GCTTGCCCCTGACCTTCATCAAGAGGAACGAG
[SQ]_M13_core_12	GTAAATTGAACGGTGTACAGACACCGAA
[SQ]_M13_core_13	CTGACCAACTTACCGTAACA
[SQ]_M13_core_14	GACAAGAAAGCTGCTCATTCAGAAATCTAC
[SQ]_M13_core_15	TAGGCTGGTGACGAGAAACACCAGGCTCATTA
[SQ]_M13_core_16	TCAGTACCAATAGGTTTGGACAGATGGGCTT
[SQ]_M13_core_17	TAGGAATAAGGACGTTGGGAAGAATGAATAAG
[SQ]_M13_core_18	TAACGCCCGATTTTAAGAACTGAACGAGTA
[SQ]_M13_core_19	GAGATGGTTTTTTTTTGGGGTTTTGC
[SQ]_M13_core_20	GTTAATAAGAAAGATTCATCAGTTTAGACT
[SQ]_M13_core_21	TACCAGTCCCACATTCAACTAATGGAAGTTTT
[SQ]_M13_core_22	ACCTTATGAAAAGGAATTACGAGGAAAACCAA
[SQ]_M13_core_23	CCTCAAGAGAAGGATTTTTTTTTTAATCATTGTGAATT
[SQ]_M13_core_24	TGCTTTAAGGGTAATAGTAAAATGTTGAGATT
[SQ]_M13_core_25	ATAAATCGAGGCTTTTGCAAAACAGATACA
[SQ]_M13_core_26	AGATTACCCTGACTATTATAGTTTACCAGACGACGATACATAGT
[SQ]_M13_core_27	AAGAGCAACATGAATGGAAAG
[SQ]_M13_core_28	GGATAGCATATTCATTGAATCCGAAGCAAA
[SQ]_M13_core_29	GCCAGAGGACAGTTCAGAAAACGATTTTAATT
[SQ]_M13_core_30	AATAGCGAAAAAATCAGGTCTTTAAGAGGAAGCCCGAAAGGCTCAACA
[SQ]_M13_core_31	ACCTTTTTTTTTTAGCCCCCTATCCTCATTTTTTACCCTCGT
[SQ]_M13_core_32	CGGATGGCAAAGCGAACCAGACCGCCCTCAAA
[SQ]_M13_core_33	TGCTGTAACTTCAAATATCGCGGAATGACC
[SQ]_M13_core_34	CAGAAGCATTTTCTGTCGTGATAAATCATTTTTCAAGAAAA
[SQ]_M13_core_35	CTCCAACCTTTTGATAAGAGGTTTTGACCA
[SQ]_M13_core_36	CGAGCTTCTTAGAGCTTAATTGCTTGATTCCC
[SQ]_M13_core_37	GCTTTCCAGTTTGCATCAAAA
[SQ]_M13_core_38	TGAAAAGGGAACGAGTAGATTTAGCATTTTTG
[SQ]_M13_core_39	GCAAAGAATAGTAGTAGCATTATTCCATATAACAGTGAATATAA

[SQ]_M13_core_40	TGTTTTAAATATGCAATTTTTTTAAGCTTGCATGCCTGC
[SQ]_M13_core_41	TTAGATAATATTTTCATTTGGGCATAAAGC
[SQ]_M13_core_42	AATTCTGCTGGCATCAATTCTACTAATTAGCAAAATTAAGGGATAAAA
[SQ]_M13_core_43	AGTTTCAACATCCATTTTCGCTTCTGGACGTT
[SQ]_M13_core_44	GTAAAACGACTTTTTTTGGTGTCTGGA
[SQ]_M13_core_45	CAACGCAACAATAAAGCCTCAGAGGCGCGAGC
[SQ]_M13_core_46	TAAATCGTTTTGCGGGAGAAGCTCAAAAGG
[SQ]_M13_core_47	CAGCCAGCTTTACAGGCAAG
[SQ]_M13_core_48	ATCTACAGCTGATAAATTAATGATGTGTAGGTAAAGATCTTTATTT
[SQ]_M13_core_49	ATTTTTAGAACCCTCATTTTTTCCCGTCGGATTCTCCG
[SQ]_M13_core_50	GTGAGAAATATTCAACCGTTCTAAAGGCTA
[SQ]_M13_core_51	TGAGTACCGGAGAGTTTTGTTAAAATTCAACA
[SQ]_M13_core_52	TTAAATGTGATTTTTTTATGCAATGCC
[SQ]_M13_core_53	TCAGGTCCGATGAACGGTAATCGTAAAACT
[SQ]_M13_core_54	TTGTAAACGTTTTTTTGAGAG
[SQ]_M13_core_55	TCCAGAGCCTAATTTGCCAGTTACAAATAAGAAACGATACATAAAA
[SQ]_M13_core_56	GAGCGCTACTTTACAGAGAGAATATTTTTG
[SQ]_M13_core_57	TTTAACGTCATTAGGTTTTGAA
[SQ]_M13_core_58	ACAGGGACTGAACAAAGTCAGACAATAGCT
[SQ]_M13_core_59	TTTTATCCTCCCGATTTTATAGCAGCATATCA
[SQ]_M13_core_60	ACCGAGGAAGAAACAATGAAATAGGGGTAATT
[SQ]_M13_core_61	GAGAGATAACTTTTTTTAGCAAGCCGT
[SQ]_M13_core_62	ATCTTACCCGAACAAAGTTACCTGGCAACA
[SQ]_M13_core_63	TAAGAGCAAACGCAATAATAACGGGTTAGCAA
[SQ]_M13_core_64	ACCAAGTACCGCACTCTTTTTTGAGTTAAGCCCAATAA
[SQ]_M13_core_65	ATATGGTTAATACATACATAAAGGAGAAGGAA
[SQ]_M13_core_66	GACATTCCTTATTACGCAGTATAATACC
[SQ]_M13_core_67	CAAAAGAACTTGCAGAACGC
[SQ]_M13_core_68	TATAAAATTGTCACAATCAATAGCAAAATC
[SQ]_M13_core_69	ACGTAGAATACCAGCGCCAAAGACCACCGACT
[SQ]_M13_core_70	TACCAGCAACATGTTTTTTAAGACTCAACCGA
[SQ]_M13_core_71	CACCGTAATTGGGAATTAGAGCCAGAAAATTC
[SQ]_M13_core_72	CCTTTAGGTGAATTATCACCGTAAAAGGGC
[SQ]_M13_core_73	TTGAGGGAGGTTTTTTTACAAATTCT
[SQ]_M13_core_74	ACCAGTAACCAATGAAACCATCACCCTCAG
[SQ]_M13_core_75	TGAGCCATTCAGTAGCGACAGAATCCACCACC
[SQ]_M13_core_76	CATTAAAGCGTCAGACTGTAGCGCCTTTTCAT
[SQ]_M13_core_77	AGCCTGTTTAGTATCATTTTTTTATTGACGGAAATTATT
[SQ]_M13_core_78	GAGCCGCCCTCCCTCAGAGCCGCCGATAGCAG
[SQ]_M13_core_79	CAGGTCATCACCGGAACCAGAGCAAGTTTG
[SQ]_M13_core_80	CGCAGTATTCACAAACAAATAATATTAGCGTTTGCCATGTTTTC
[SQ]_M13_core_81	ATCGGCATTTTGCTTAGGTTG

[SQ]_M13_core_82	AACCGCCAGCCGCCACCAGAACAATAAGTT
[SQ]_M13_core_83	GGAACCGCGCCAGCATTGACAGGACATGGCTT
[SQ]_M13_core_84	AATCAAAAGACGATTGGCCTTGATCTCTGAATTTACCGTTCTGAGACT
[SQ]_M13_core_85	TCGGAACCACAGGAGTGTACTGGTCACCACCA
[SQ]_M13_core_86	TAAGAGGCCAGTAAGCGTCATAGGTTGAGG
[SQ]_M13_core_87	TTAACGGACAGTTAATGCCCCCAATAGGTG
[SQ]_M13_core_88	TTGATGATTATTATTCTGAAACATCCGTCGAG
[SQ]_M13_core_89	CCCTCAGATATAAGTATAGCCCGGTGCCTATT
[SQ]_M13_core_90	CTGAGTTCAGGGATAGCAAGCCCAGGCGGATAAGTGGAAAGTAT
[SQ]_M13_core_91	TATCACCAACCGCCACCCTCAGCATTCCAC
[SQ]_M13_core_92	AGGGTTGAGCCACCACCCTCATTTTTCGTCACCAGTACAACTGTATGG
[SQ]_M13_core_93	TGAATTTTACTACAACGCCTGTAGAACCGCCA
[SQ]_M13_core_94	AGACAGCTCGTCTTTCCAGACGAAGGAATT
[SQ]_M13_core_95	TTTCTTGCCTTTAATTGTATCGTAGAAAGGAACAACTATTAGTAAA
[SQ]_M13_core_96	GCGAATAAAGGCTCCAAAAGGAAAACAGCT
[SQ]_M13_core_97	TGATACCCCCACGCATAACCGATATATTCG
[SQ]_M13_core_98	AAGAATACGTGGCACAGACAATAACTGATAGCCCTAAAAGGTGAGG
[SQ]_M13_core_99	TAAAGCATGCAGAAGATAAAACAGACATCG
[SQ]_M13_core_100	CCATTAAAAATTTTTACATTGG
[SQ]_M13_core_101	CGGTCAGGAGCCAGCAGCAAATAAGGAATT
[SQ]_M13_core_102	TCAAACCGTCTGAATTTTAACCACCACACCTT
[SQ]_M13_core_103	GATAATACGCAAATCAACAGTTGAGAAAAATC
[SQ]_M13_core_104	GCTGAACCTCTTTTTTTGTAGAAGAAC
[SQ]_M13_core_105	GAGGAAGCTAATAGATTAGAGCCATTATCA
[SQ]_M13_core_106	GTCAGTTGATTTGAGGATTTAGAACCGAACGT
[SQ]_M13_core_107	GATTAGTAATAACATCTTTTTTCCTCAATCAATATCTG
[SQ]_M13_core_108	TGATGGCATTAAAAGTTTGAGTAACGTCAATA
[SQ]_M13_core_109	TTGGATTTATTAAATCCTTTGCGTATTA
[SQ]_M13_core_110	GACTTTACAACAGGAGGCCG
[SQ]_M13_core_111	TTTTGCGTCATCATATTCCTGATCAGATGA
[SQ]_M13_core_112	TATTAATTATTCATCAATATAATCTAAAGAAA
[SQ]_M13_core_113	AAAGGACAGAGCGGTTTTACAACTCGATACTT
[SQ]_M13_core_114	AATACCAAATTTTCAGGTTTAACGTTATCAGA
[SQ]_M13_core_115	TTATTCACACGTAAAACAGAAACTGATTGT
[SQ]_M13_core_116	CTGAATAATGTTTTTTTACGTGGCGAG
[SQ]_M13_core_117	ATATACAAACGGATTCGCCTGAAATAACCT
[SQ]_M13_core_118	TTGCGTAGGTTACAAAATCGCGCAGGAAACAG
[SQ]_M13_core_119	ATTATTTGTTTCAATTACCTGAGCTAACAATT
[SQ]_M13_core_120	AGAGCTTGACGGGGAATTTTTTTAACCTACCATATCAAA
[SQ]_M13_core_121	AGCGATAGTCAATATATGTGAGTGTTGCTTTG
[SQ]_M13_core_122	TCAATAGATTACCTTTTTTAATGAGGCGAA
[SQ]_M13_core_123	GGTTATATAGGTCTGAGAGACTCAAAATTAATTACATTAAAAGA

[SQ]_M13_core_124	AGATGATGAATGCCCGAGATA
[SQ]_M13_core_125	TGCTTCTTCCCTTAGAATCCTTCAAATATA
[SQ]_M13_core_126	TACATAAACTTAGATTAAGACGCTATCGCAAG
[SQ]_M13_core_127	TCATTTGATGAATTTATCAAAATCATAACTATATGTAAATCTAGAAAA
[SQ]_M13_core_128	TAAGGCGTCGCGAGAAAACTTTTTGAAAACAT
[SQ]_M13_core_129	ATAATTAGCTGATGCAAATCCAGAGAAGAG
[SQ]_M13_core_130	TTTTAGTTGAAATACCGACCGTTTAACAAC
[SQ]_M13_core_131	ACAAAGAATAAATAAGAATAAACACAACAGTA
[SQ]_M13_core_132	CAAAAGGTTTGAGAATCGCCATATGTGATAAA
[SQ]_M13_core_133	GCCTGTGACGACGACAATAAATATAAAGCCAACGCTCCGGAATC
[SQ]_M13_core_134	GCCAACAAATAAGAGAATATAAAATAATAT
[SQ]_M13_core_135	GGGCTTAAAAAGTAATTCTGTCCATTATCAACAATAGATAGGTATTAA
[SQ]_M13_core_136	CAAGAACGAGTCCTGAACAAGAAAAGTACCGA
[SQ]_M13_core_137	CCCATCCTCGGCTGTCTTTCCTATTACCGC
[SQ]_M13_core_138	GCCTTAGAGGCGTTTTAGCGAATTTCATCGTAGGAATCTATCATTC
[SQ]_M13_core_139	GCCCAATGTATTCTAAGAACGCAATCAAGA
[SQ]_M13_core_140	TTAGTTGATCTTACCAACGCTAACGAGCGT
[SQ]_M13_core_141	ATGTCAATCATATGTACCCCGGTTTGTATAAGCAAATATTAAATCA
[SQ]_M13_core_142	AGCTTTCATCGCATTAAATTTTTGTTTAAA
[SQ]_M13_core_143	GCTCATTTCGCGTCTGGCCTTCTAATGGGA
[SQ]_M13_core_144	GACAGTATAACGGCGGATTGACCGCTGTAGCC
[SQ]_M13_core_145	TAGGTCAATCTGCCAGTTTGAGTGTTGGGA
[SQ]_M13_core_146	TGGGAACACGGCCTCAGGAAGATCCAAAGCGC
[SQ]_M13_core_147	GGCGATTAATTCAGGCTGCGCAACGGGACGAC
[SQ]_M13_core_148	CAGTCACGTGCCGGAAACCAGGGCACTC
[SQ]_M13_core_149	AGGGCGAGGCGAAAGGGGGATGAGCTGTTT
[SQ]_M13_core_150	CATTCGCCAGTTGGGTAACGCCAGGGTACCGA
[SQ]_M13_core_151	TAAAGCCTTCGTAATCATGGTCATTGCTGCAA
[SQ]_M13_core_152	TCACATTTCTAGAGGATCCCCGGGTTTTCC
[SQ]_M13_core_153	CCTGTGTATACGAGCCGGAAGCCAGTGAGA
[SQ]_M13_core_154	GCTCGAATGGGGTGCCTAATGAGTGTATTGGG
[SQ]_M13_core_155	AGGTCGACAATTGCGTTGCGCTCAATCGGCCA
[SQ]_M13_core_156	GGTCCACGTGGTTTTTCTTTTCACATAAAGTG
[SQ]_M13_core_157	TCCTGTTGGAGAGGCGGTTTGCGAGCTAAC
[SQ]_M13_core_158	GGGTTGATCGGCAAAATCCCTTCCAGCTGCATTAATGACTGCCC
[SQ]_M13_core_159	CGGGCAAGCCCTGAGAGAGTTGCGAAAAAC
[SQ]_M13_core_160	CGCCAGGGCTGGTTTGCCCCAGCATATTAAAG
[SQ]_M13_core_161	ACGCGCGGTGATGGTGGTTCCGAAAGTGTTGTTCCAGTTTCCCGATTT
[SQ]_M13_core_162	GGTGCCGTCTCCAACGTCAAAGGGCAGCAAGC
[SQ]_M13_core_163	GGGAGCCGGAACAAGAGTCCACGGCGAAAA
[SQ]_M13_core_164	CGTCTATCAAATCAAGTTTTTTCGGTCACG
[SQ]_M13_core_165	AACGTGGAAAAGCACTAAATCGGAAGGAGCGG

[SQ]_M13_core_166	CTTTGACGGCGCTGGCAAGTGTAGGGGGTCGA
[SQ]_M13_core_167	ATTAAATTTCCTCGTTAGAATAGGGAAGAAAGCGAAACCCTAAA
[SQ]_M13_core_168	CTGCGCGCTACAGGGCGCGTACCTGAGAAG
[SQ]_M13_core_169	GCGCTAGGAGCACGTATAACGTGCGGGATTTTAGACAGGAACTTCTTT
[SQ]_M13_core_170	GTAGCAATACGGTACGCCAGAATCTATGGTTG
[SQ]_M13_core_171	TGTTTTTGTCCATCACGCAAATAATATCCA
[SQ]_M13_core_172	CAGATTCATTTTGACGCTCAATTATCGGCCTTGCTGGTTAACCGTT
[SQ]_M13_core_173	GAACAATTCATGGAAATACCTACACCAGTC
[SQ]_M13_core_174	ACACGACATAGAACCCTTCTGACCTGAAAG

Supplementary Table 2. Edge staples of M13mp18 square DNA origami tiles.

Name	Sequence
[SQ]_M13_edge_1	GCATTTTCGAGCCAGTTGTAATTTAGGCAGAGCG
[SQ]_M13_edge_2	CCTAAATTTAATGGTTTAATTTCATCTTCTGACA
[SQ]_M13_edge_3	CATCGGGAGAAACAATGTAACAGTACCTTTTATA
[SQ]_M13_edge_4	AGAAGGAGCGGAATTAGAACAAAGAAACCACCCG
[SQ]_M13_edge_5	CTATTACGCCAGCTTCGGTGCGGGCCTCTT
[SQ]_M13_edge_6	CAATTCCACACAACGAAATTGTTATCCGCT
[SQ]_M13_edge_7	CGTGAACCATCACCCAGGGCGATGGCCCAC
[SQ]_M13_edge_8	CGCTTAATGCGCCGTAACCACCACACCCGC
[SQ]_M13_edge_9	CCAAATCAACGTAACAACCGGATATTCATTACGT
[SQ]_M13_edge_10	CAACATTATTACAGGTAAACGAACTAACGGAAAA
[SQ]_M13_edge_11	GTACCTTTAATTGCTCAGGTCAGGATTAGAGACA
[SQ]_M13_edge_12	AATAACCTGTTTAGCTCATTTCGCAAATGGTCAC
[SQ]_M13_edge_13	ACCGCCACCCTCAGGTACTCAGGAGGTTTA
[SQ]_M13_edge_14	CAGTGCCCGTATAAGGTCAGTGCCTTGAGT
[SQ]_M13_edge_15	AGGCCGGAAACGTCGCACCATTACCATTAG
[SQ]_M13_edge_16	GGAATAAGTTTATTGAAACGCAAAGACACC

Supplementary Table 3. Reinforcing edge staples of M13mp18 square DNA origami tiles.

Name	Sequence
[SQ]_M13_edge_17	TCGTCACCCTCAGCAGGGCCGCTTTTGCGGGAAT
[SQ]_M13_edge_18	ACGTAATGCCACTACGATTAAACGGGTAAAATGA
[SQ]_M13_edge_19	AAATCCGCGACCTGCTCTGATAAATTGTGTCGAC
[SQ]_M13_edge_20	TATGACCCTGTAATACGTTGTACCAAAAACATAA
[SQ]_M13_edge_21	AATCACCATCAATATGAGGCCGGAGACAGTCAGA
[SQ]_M13_edge_22	GAGCAAACAAGAGAATATTGCCTGAGAGTCTGAT
[SQ]_M13_edge_23	TATTTATCCCAATCCAAAATAAACAGCCAT
[SQ]_M13_edge_24	ATTAACTGAACACCAGCGCATTAGACGGGA
[SQ]_M13_edge_25	AAGTAAGCAGATAGCGAAGCCCTTTTTAAG
[SQ]_M13_edge_26	GATCTAAAGTTTTGCCTCATAGTTAGCGTA
[SQ]_M13_edge_27	AAATCTCCAAAAAAATAATTTTTTCACGTT
[SQ]_M13_edge_28	GACAACAACCATCGGATAGTTGCGCCGACA
[SQ]_M13_edge_29	AGTCTTTAATGCGCGATTTTTGAATGGCTATTAT
[SQ]_M13_edge_30	AACAGTGCCACGCTGATATTAACACCGCCTGCGC
[SQ]_M13_edge_31	TTAGGAGCACTAACAAGTTATCTAAAATATCTCC
[SQ]_M13_edge_32	AGAAACCAATCAATAATAATTTACGAGCATGTCG
[SQ]_M13_edge_33	ATAGAAGGCTTATCCGAGCAAGCAAATCAGATCG
[SQ]_M13_edge_34	TACAATTTTATCCTGACTATTTTGCACCCAGCCC
[SQ]_M13_edge_35	CAAAAACAGGAAGATGATAATCAGAAAAGC
[SQ]_M13_edge_36	CCATCAAAAATAATTTTTAACCAATAGGAA
[SQ]_M13_edge_37	CATCGTAACCGTGCCGTTGGTGTAGATGGG
[SQ]_M13_edge_38	GAGTAAAAGAGTCTATAATCAGTGAGGCCA
[SQ]_M13_edge_39	AACAGGAAAAACGCATTACCGCCAGCCATT
[SQ]_M13_edge_40	TCTGGCCAACAGAGCAGTAATAAAAGGGAC

Supplementary Table 4. Core staples of p3548 regular hexagonal DNA origami tiles.

Name	Sequence
[HE]_p3548_core_1	GTAAACTTGGTCCTATCTCAGCGATCAACTACGA
[HE]_p3548_core_2	GTGTAGATTGTCTATTTCTTTTTTTTAAAAAGGATCGAAAACTC
[HE]_p3548_core_3	TACGGGATACCGCGAGACCCACAATTGTTG
[HE]_p3548_core_4	TACAGGCACTCCATCCAGTCTATTGCTCACCGGCTCCAGATCCCCGTC
[HE]_p3548_core_5	TTTGCAAGGGATTTTTGCCTGACTTTATCAG
[HE]_p3548_core_6	CAATAAACCTGCAACTTTATCCGCTCGTGGTGTCACGCTCCAACGATC
[HE]_p3548_core_7	TGGTCCAGCCTTTTGGTGGGATCC
[HE]_p3548_core_8	CCGGGAATTGCGCAACGTTGTTGTTGTGCA
[HE]_p3548_core_9	GGCAAACAAACCATTTTTTTTTCCGAGCGCAGAAG
[HE]_p3548_core_10	TCACTCATTTACATGATCCCCCATGCCATTGC
[HE]_p3548_core_11	GGAAGTCACCTTTTGTTCCGTCGT
[HE]_p3548_core_12	TTGGTATGGCTTCTTTTTTTTTGTGAGCAAAAACA
[HE]_p3548_core_13	AAAAAGCGAAGTAAGTTGGCCGTCATGCCATCCGTAAAAGTCATTCTG
[HE]_p3548_core_14	AAGGCGAGGGTTATGTTTGCTCATTCAGCATC
[HE]_p3548_core_15	AACGTTCTCGCCACATAGTTTTTTTTTCTCTTACTGCAGTGTTA
[HE]_p3548_core_16	ATACCTGTCCGCATAGCTCACGCTGTGCACGAAC
[HE]_p3548_core_17	GGCTGTGTAGGTATCTCATTTTTTTTAAGATACCAGCGCTCAAG
[HE]_p3548_core_18	CCCCCGTATCGTCTTGAGTCCATGGCCTAA
[HE]_p3548_core_19	TCGGAAAAAGAGTTCTTGAAGTGGACCCGGTAAGACACGACCAAGCTG
[HE]_p3548_core_20	AGCAAAAGGGCGAAATTGTTCGCTCTTATCGC
[HE]_p3548_core_21	CACTGGCAATGTAGGCGGTGCTACAGAGTTGGTAGCTCTTTTTTTTTG
[HE]_p3548_core_22	GAGGTGCAGCTTTTTGAGCAATCA
[HE]_p3548_core_23	CTACGGCCGCTCTGCTGAAGCCGAAAAAAA
[HE]_p3548_core_24	GGGGATAACGCAGTTTTTTTTTGGATTAGCAGAGC
[HE]_p3548_core_25	ACGTTAAGCAGCAGATTACGCGCAAGTTACCT
[HE]_p3548_core_26	GGATCTCGACGCTCAGTGGAACTTCACCTAGATCCTTAAGTATATATG
[HE]_p3548_core_27	ACTCCAACGTCCCCACTACGTGAACCATCGGAAC
[HE]_p3548_core_28	AGCACTAAATCACCCTAATTTTTTTTAAGAATAGACCCAATAGG
[HE]_p3548_core_29	CCTAAAGCCGGCGAACGTGGCGGCCGCTAC
[HE]_p3548_core_30	GCCTCTTCCCGCCGCGCTTAATGCAGAAAGGAAGGGAAGATGCCGTAA
[HE]_p3548_core_31	TCCCCGAAGGCAAAATTGTCGAGGAAGCGAAA
[HE]_p3548_core_32	GGAGCGGGCGCGTAACCACCACACGCTATTACGCCAGCTGGGGTAACG
[HE]_p3548_core_33	CGCTGCGCTATTTTTTCCGATGTA
[HE]_p3548_core_34	AGGGCGCGTTGGGAAGGGCGATTGTAAAAC
[HE]_p3548_core_35	TTTAGAAAAATAATTTTTTTTTAGTGTAGCGGTCA
[HE]_p3548_core_36	TCGAGGTCTTCCCAGTCACGACGTCGGTGCGG
[HE]_p3548_core_37	ATTCGAGCCATTTTAAGTTGCGAA
[HE]_p3548_core_38	AGGGGGATGTGCTTTTTTTTTTCAGCGCAGCAATT
[HE]_p3548_core_39	GACGGCCCGAATTGGGTACCGGGCTCATCTGTCTGTAAACAAGACTGG
[HE]_p3548_core_40	CCAGGGTTGACGGTATTGGCATCACCATCGGG

[HE]_p3548_core_41	CGGACAAACGTGACTCGCTTTTTTTGCAATGGTGAGCCCCCCC
[HE]_p3548_core_42	GAATAGTGTATTACGGGATAATACCGTCGGGGCG
[HE]_p3548_core_43	AAAACTCGATGTAACCCACTCGCACGGAAA
[HE]_p3548_core_44	TCTCATGAAAGGGAATAAGGGCGATGCACCCAACTGATCTCATTGGAA
[HE]_p3548_core_45	TTTTACTTGCAAAATGCCGCAAAAGCGGATACATATTTGACGCACATT
[HE]_p3548_core_46	TGTTGAATGAAGCATTTATCAGGTAAGCGT
[HE]_p3548_core_47	CCGAAATCAAGTGCCACCTAAATTGGTTATTG
[HE]_p3548_core_48	TAATATTCAGCTCATTTTTTAACGAGATAGGGTTGAGTAAAGAACGTG
[HE]_p3548_core_49	CCTTTAGTGAGTAGCTGTTTCCTGTGCATAAAGT
[HE]_p3548_core_50	GCCGGAAGTGAAATTGTTTTTTTTTTTTCCTGCAGCCGACGTGT
[HE]_p3548_core_51	GTAAAGCTAATTGCGTTGCGCTGCTTCCTC
[HE]_p3548_core_52	AGGCGGTATATTGGGCGCTCTTCCCACTGCCCGCTTTCCAACATACGA
[HE]_p3548_core_53	ATACCTTCCCGAACATTCCACACAGTCGGGAA
[HE]_p3548_core_54	ACCTGTCGGGAGAGGCGGTTTGCGATACGGTTATCCACAGAAAAGGCC
[HE]_p3548_core_55	CGCGGTGCCATTTTCATGTCCTTG
[HE]_p3548_core_56	GCTCACTGCGAGCGGTATCAGCAGGCCGCG
[HE]_p3548_core_57	AAGTCTGATGCACTTTTTTTTTGAATCGGCCAACG
[HE]_p3548_core_58	TCAGAGGTGCCAGGAACCGTAAAATCACTCAA
[HE]_p3548_core_59	TTGCTGGCATCACAAAAATCGAGCGTTTCCCCCTGGAGCCGCTTACCG
[HE]_p3548_core_60	GGCGTGAAAATAGATCCCTGGCTTAAATCAACGA
[HE]_p3548_core_61	CAAATCTATCGGGTGGATAGTTATAGGAAA
[HE]_p3548_core_62	GAAGAGTGGTCCGCTCCAACCAGGGGAAAAAAGAAGTTATGCGGACTA
[HE]_p3548_core_63	GCAAGATAGCCAGCACGAAACGCAGTGCAATTCGACGTCAGTATCAAG
[HE]_p3548_core_64	AAAGCAAGGAAAAAAGCGTGCGGAGAAGGC
[HE]_p3548_core_65	CACAATGATATCCGAAAAAAGAGGTGTAGTCG
[HE]_p3548_core_66	CCGTAAAAGTGGCAGGACTTCTCCGGGGGATCCACTACAGCTTTTGTT

Supplementary Table 5. Edge staples of p3548 regular hexagonal DNA origami tiles.

Name	Sequence
[HE]_p3548_edge_1	AAGCGTGGCGCTTTCTCCTTTCTCCCTTCGGGGA
[HE]_p3548_edge_2	GCCTTATCCGGTAACTTCAGCCCGACCGCTGCAC
[HE]_p3548_edge_3	CTTTTCTACGGGGTCTAAGAAGATCCTTTGATAG
[HE]_p3548_edge_4	GTTTTAAATCAATCTATTAAATTAAAAATGAATC
[HE]_p3548_edge_5	ACCAGCTCAGAAAACGTTCGTACCCACGTA
[HE]_p3548_edge_6	GACTCACTATAGGGAGTGAGCGCGCGTAAT
[HE]_p3548_edge_7	CTTGACGGGGAAAGGGAGCCCCCGATTTAG
[HE]_p3548_edge_8	TATCAGGGCGATGGAAAGGGCGAAAAACCG
[HE]_p3548_edge_9	CTTAATCAGTGAGGCACTGACAGTTACCAATGCA
[HE]_p3548_edge_10	CCCAGTGCTGCAATGAGGGCTTACCATCTGGCGA
[HE]_p3548_edge_11	CCTCCGATCGTTGTCAGGTTAGCTCCTTCGGTAC
[HE]_p3548_edge_12	TGGTGAGTACTCAACCGATGCTTTTCTGTGACAA
[HE]_p3548_edge_13	CCGCGGTGGAGCTCGTTCTAGAGCGGCCGC
[HE]_p3548_edge_14	AAAAACTCTCGTACAGGAAGCTGTACTGCG
[HE]_p3548_edge_15	GCAGAGAGCTCAAAGCCAGGGAAAAAAGAT
[HE]_p3548_edge_16	AAAGTCACCAATCGTGAGCGATTGACAGGA
[HE]_p3548_edge_17	GGCGTAATCATGGTCAGGTTAATTGCGCGCTTAA
[HE]_p3548_edge_18	GTGAGCTAACTCACATCTGGGGTGCCTAATGATA
[HE]_p3548_edge_19	CCGCCCCCCTGACGAGCGTTTTTCCATAGGCTTG
[HE]_p3548_edge_20	CTCCTGTTCCGACCCTAGCTCCCTCGTGCGCTTC
[HE]_p3548_edge_21	CAAGAGTCCACTATTGTTGTTCCAGTTTGG
[HE]_p3548_edge_22	AATTTTTGTTAAATTTGTTAAAATTCGCGT
[HE]_p3548_edge_23	TTGAGATCCAGTTCTCAAGGATCTTACCGC
[HE]_p3548_edge_24	TTGCCCGGCGTCAAGCGGCGACCGAGTTGC

Supplementary References

[1] Blake, P., Hill, E.W., Castro Neto, *et al*. 2007. ***Appl. Phys. Lett. 91*** (2007).

[2] Aspnes, D.E., Studna., A.A., ***Phys. Rev. B*** **27**, 985-1009 (1983).

[3] Malitson, Ian H. ***J. Opt. Soc. Am.*** **55**, 1205-1208 (1965).

[4] Tan, C. Z. ***J. Non-Cryst. Solids*** **223**, 158-163 (1998).

[5] Song, J., Arbona, J.M., Zhang, Z., *et al.* ***J. Am. Chem. Soc.*** **134**, 9844-9847 (2012).

[6] Barcons Ruiz, D., Herzig Sheinfux, H., Hoffmann, R., *et al.* ***Nat. Commun.*** **13,** p.6926 (2022).
